# Maturation signatures of conventional dendritic cell subtypes in COVID‐19 suggest direct viral sensing

**DOI:** 10.1002/eji.202149298

**Published:** 2021-10-01

**Authors:** Laura Marongiu, Giulia Protti, Fabio A. Facchini, Mihai Valache, Francesca Mingozzi, Valeria Ranzani, Anna Rita Putignano, Lorenzo Salviati, Valeria Bevilacqua, Serena Curti, Mariacristina Crosti, Maria Lucia Sarnicola, Mariella D'Angiò, Laura Rachele Bettini, Andrea Biondi, Luca Nespoli, Nicolò Tamini, Nicola Clementi, Nicasio Mancini, Sergio Abrignani, Roberto Spreafico, Francesca Granucci

**Affiliations:** ^1^ Department of Biotechnology and Biosciences University of Milano‐Bicocca Milan Italy; ^2^ National Institute of Molecular Genetics “Romeo ed Enrica Invernizzi” Milan Italy; ^3^ Pediatric Department and Centro Tettamanti‐European Reference Network PaedCan, EuroBloodNet MetabERN‐University of Milano‐Bicocca‐Fondazione MBBM‐Ospedale, San Gerardo Monza Italy; ^4^ ASST san Gerardo Hospital, School of Medicine and Surgery University of Milano‐Bicocca Milan Italy; ^5^ Laboratory of Medical Microbiology and Virology Vita‐Salute San Raffaele University Milan Italy; ^6^ IRCCS San Raffaele Hospital Milan Italy; ^7^ Department of Clinical Sciences and Community Health University of Milano Milan Italy; ^8^ Institute for Quantitative and Computational Biosciences University of California Los Angeles

**Keywords:** COVID‐19, dendritic cells, single cell transcriptomics

## Abstract

Growing evidence suggests that conventional dendritic cells (cDCs) undergo aberrant maturation in COVID‐19, which negatively affects T‐cell activation. The presence of effector T cells in patients with mild disease and dysfunctional T cells in severely ill patients suggests that adequate T‐cell responses limit disease severity. Understanding how cDCs cope with SARS‐CoV‐2 can help elucidate how protective immune responses are generated. Here, we report that cDC2 subtypes exhibit similar infection‐induced gene signatures, with the upregulation of IFN‐stimulated genes and IL‐6 signaling pathways. Furthermore, comparison of cDCs between patients with severe and mild disease showed severely ill patients to exhibit profound downregulation of genes encoding molecules involved in antigen presentation, such as MHCII, TAP, and costimulatory proteins, whereas we observed the opposite for proinflammatory molecules, such as complement and coagulation factors. Thus, as disease severity increases, cDC2s exhibit enhanced inflammatory properties and lose antigen presentation capacity. Moreover, DC3s showed upregulation of anti‐apoptotic genes and accumulated during infection. Direct exposure of cDC2s to the virus in vitro recapitulated the activation profile observed in vivo. Our findings suggest that SARS‐CoV‐2 interacts directly with cDC2s and implements an efficient immune escape mechanism that correlates with disease severity by downregulating crucial molecules required for T‐cell activation.

## Graphical abstract

DC2s and DC3s show similar gene signatures in response to SARS‐CoV‐2 infections, and their activation responses are dominated by ISGs. cDC2 subtypes enhance their inflammatory properties and progressively lose antigen presenting functions as disease severity increases. DC2s and DC3s can be activated by direct viral sensing.

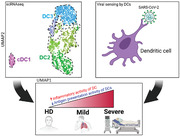

## Introduction

Clinical outcomes of COVID‐19 are highly variable. Patients may show either no or mild symptoms (such as mild fever and cough) or severe respiratory involvement requiring hospitalization. In the most severe cases, acute respiratory distress syndrome (ARDS) can develop, with high levels of inflammatory molecules in the blood [1, 2] and diffuse intravascular coagulation (DIC) [3, 4]. COVID‐19 is lethal in a non‐negligible number of cases [5]. Patients presenting severe symptoms show immune dysregulation, characterized by excessive release of type 1 and type 2 cytokines [2] and alterations of lymphoid and myeloid populations in the peripheral blood [6]. Severely ill patients, in contrast to patients with mild disease, also show alterations in both Th17 and Th1 cell activation, with defects in the acquisition of effector functions [7].

Cells of myeloid origin play a pivotal role during infections by sensing pathogens, producing inflammatory mediators, and contributing to the activation of adaptive immunity. In this context, DCs are particularly relevant, as they are specialized in antigen presentation and T‐cell priming [8]. The differences observed in the activated T‐cell compartments of patients with severe versus mild disease suggest abnormal activation in the conventional DC (cDC) compartment of patients presenting with more severe disease.

cDCs have been divided into two subtypes, cDC1s and cDC2s, originating from a common precursor (pre‐DCs) [9, 10, 11, 12]. cDC1s have a high intrinsic capacity to cross‐present antigens, due to expression of the CLEC9A c‐type lectin [13], and activate CD8^+^, Th1, and NK cells [14]. Myeloid cDC2s express various pattern recognition receptors (PRRs) and can promote a wide range of immune responses, especially CD4^+^ T‐cell responses [15]. Recently, cDC2s have been divided into two subsets, DC2s and DC3s [16, 17, 18]. DC3s have been described as a heterogeneous population that expands in inflammatory conditions [16].

Functional impairment of cDCs has been described in COVID‐19 patients, with decreased numbers in the blood [19, 20] and reduced functionality, measured in terms of cytokine production and T‐cell priming capacity [21] upon in vitro restimulation. Nevertheless, a defect in maturation upon in vitro restimulation does not necessarily indicate functional impairment, as activated DCs may not further respond to PRR agonists. No specific information is available concerning the impact of SARS‐CoV‐2 infection on the maturation of DC subtypes. A deeper understanding is crucial, given the important role of cDC subtypes in the activation and skewing of adaptive immune responses that ultimately contribute to COVID‐19 pathogenesis [22, 23]. Here, we characterized the transcriptional signatures reflecting the functional state of cDC subpopulations by high‐throughput single‐cell RNA sequencing (scRNA‐seq).

## Results

### Transcriptional signatures of circulating cDCs in COVID‐19 patients

We analyzed peripheral blood cDCs from COVID‐19 patients with severe and mild disease, according to the World Health Organization (WHO) classification, to better understand the impact of SARS‐CoV‐2 infection on cDC subtypes. Patients were enrolled from the STORM cohort (see Table S1 for clinical data of the patients) of San Gerardo Hospital in Monza, Italy.

**Figure 1:**
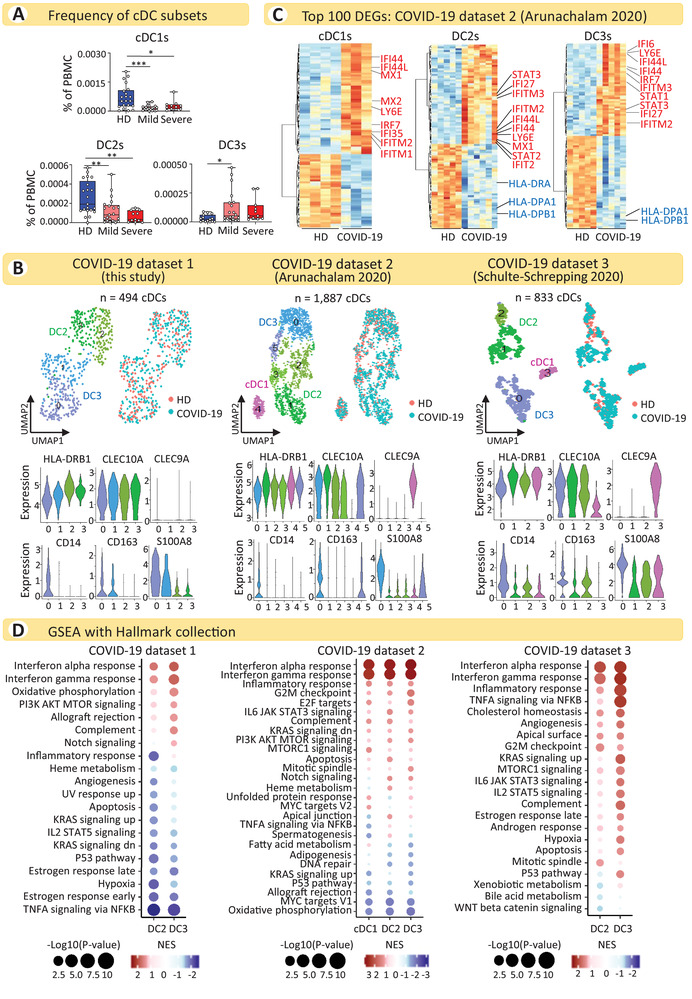
**The response of cDCs to SARS‐CoV‐2 infection is dominated by ISGs**. (A) Percentage of cDC1s, DC2s, and DC3s among PBMCs from the whole blood of COVID‐19 patients (n = 22 with mild and n = 10 with severe disease) and HDs (n = 21). Statistical significance was determined using one‐way analysis of variance (ANOVA), followed by Sidak's multiple comparison test. *p < 0.05, **p < 0.01, ***p < 0.001. Each dot represents an individual donor. (B, upper panels) UMAP representations of cDC subpopulations identified in the three COVID‐19 datasets analyzed: dataset 1 (*n* = 2 HDs, *n* = 3 COVID‐19), dataset 2 (*n* = 5 HDs, *n* = 7 COVID‐19), and dataset 3 (*n* = 21 HDs, *n* = 18 COVID‐19). Cells are colored according to cDC subpopulation and donor origin. (B, lower panels) Violin plots illustrating the expression levels of selected marker genes used for the manual annotation of cDC subtypes. (C) Heatmaps showing the top 100 DEGs for each cDC subset comparing COVID‐19 patients and HDs from dataset 2. Differential expression analysis was performed using pseudo‐bulk counts and, for each DC subset, only donors with at least 10 cells were retained (cDC1s: *n* = 4 HDs, *n* = 3 COVID‐19; DC2s: *n* = 5 HDs, *n* = 6 COVID‐19; DC3s: *n* = 5 HDs, *n* = 5 COVID‐19). Selected upregulated genes (ISGs) are marked in red and downregulated genes in blue. Ribosomal protein (RP) genes were removed from the top 100 DEGs (see Table S2 for the full list of DEGs). (D) GSEA of DEGs using the Hallmark collection for the three COVID‐19 datasets. For each dataset, the top 15 pathways in each cDC subset were selected and consolidated across all DC subsets in a single dot plot. NES, normalized enrichment score.

Amongst CD11c^+^MHCII^+^ peripheral blood mononuclear cells (PBMCs), cDC1s were identified as CLEC9A^+^ and cDC2s were identified among the CD1c^+^FcεRIα^+^ cells, excluding cells expressing markers for T or B lymphocytes (CD3 and CD19, respectively) or monocytes (CD88 and CD89) [16]. CD14 was included in the analysis to identify DC3s [17] (Supplementary Fig. 1 for gating strategies). Consistent with previous studies, we found a decreasing trend in the frequency of cDC1s and DC2s [19, 20] and an increasing trend in DC3s in the blood of COVID‐19 patients relative to that of healthy donors (HDs) (Fig. 1A). The decreased number of cDCs in circulation may be due to both their recruitment to the respiratory tract and to apoptotic death caused by exposure to an inflammatory environment or direct viral encounter.

We systematically characterized the transcriptional response of cDCs to SARS‐CoV‐2 infection by analyzing three different single‐cell transcriptomic datasets, two that were publicly available and one that was newly generated. Analysis of three independent datasets allowed us to identify consistently altered signaling pathways, minimizing the effects of possible biases in the single datasets.

The new dataset (dataset 1) [24] was generated using a droplet‐based single‐cell platform (10X Chromium) and contains scRNA‐seq data of CD11c^+^MHCII^+^ cells isolated from PBMCs of three COVID‐19 patients (two with mild and one with severe disease) and two HDs (Table S1). The second dataset [22] (dataset 2) contains cellular indexing of transcriptomes and epitopes by sequencing (CITE‐seq) data of PBMCs and enriched DCs obtained from seven COVID‐19 patients (three with mild and four with severe disease) and five HDs, and the third dataset [25] (dataset 3) contains scRNA‐seq data of PBMCs obtained from 18 COVID‐19 patients (8 with mild and 10 with severe disease) and 21 HDs.

Single‐cell data from datasets 1 and 2 were first visualized using non‐linear dimensionality reduction through uniform manifold approximation and projection (UMAP) and graph‐based clustering algorithms (Supplementary Fig. 2A, 3A). Clusters containing myeloid DCs were identified based on the expression of markers that discriminate cDC2s and cDC1s from all other cell populations. Specifically, *CD1C*, *FCER1A*, and *CLEC10A* were used to identify cDC2s, whereas *CLEC9A* was used to identify cDC1s (Supplementary Fig. 2B, 3B). Myeloid DCs already annotated by the authors were considered for dataset 3 [25]. Clusters corresponding to myeloid DCs in the three datasets were re‐clustered in further iterations to separate cDC1s from cDC2s, to discriminate cDC2 subpopulations and to exclude possible contaminants or doublets. Specifically, DC3s were distinguished from DC2s based on the expression of *CD14*, *CD163*, and *S100A8*. This approach allowed us to clearly identify cDC subsets (Fig. 1B, Supplementary Fig. 3C,D,E and Supplementary Fig. 4A).

Next, we aggregated cell‐level counts into sample‐level pseudo‐bulk counts, mitigating single‐cell mRNA measurement noise, to unravel the transcriptional response of each cDC subset during SARS‐CoV‐2 infection and identified differentially expressed genes (DEGs) between COVID‐19 patients and HDs (Table S2). The low numbers of cDC1s allowed their analysis solely in dataset 2.

Comparison of the expression profiles of COVID‐19 patients with those of HDs showed most of the genes upregulated in COVID‐19 to be interferon (IFN) stimulated genes (ISGs) in all cDC subsets (Fig. 1C and Supplementary Fig. 5A,B). On the other hand, genes encoding MHCII molecules were among the most significantly downregulated genes in cDCs from COVID‐19 patients (Fig. 1C), indicating that these cells have an impaired antigen presentation capacity.

We performed gene set enrichment analysis (GSEA) to better understand the biological signaling pathways that are differentially regulated in cDCs from COVID‐19 patients relative to HDs using two gene sets: the Hallmark collection from the Molecular Signatures Database (MSigDB) and the literature‐derived Blood Transcription Modules (BTMs) [26] (Table S3). As expected, maturation was dominated by ISGs in all cDC subtypes from the three datasets, consistent with the identified DEGs, whereas we were unable to detect the upregulation of signatures containing classical activation markers and cytokines for T‐cell priming (Fig. 1D and Supplementary Fig. 6A). Along with the IFN‐induced pathways, IL‐6 pathways (IL‐6‐JAK‐STAT3 and PI3K‐AKT‐mTOR [27]) were consistently upregulated in cDC2s in all datasets (Fig. 1D). This is in accordance with the relevance of IL‐6 in COVID‐19 pathogenesis and the expansion of activated Th17 cells in COVID‐19 patients [28].

### Circulating cDCs in patients with urinary tract bacterial infections and vaccinated individuals

The absence of a conventional maturation signature (absence of upregulation of genes encoding MHCI‐II, costimulatory molecules, and cytokines) in circulating DCs prompted us to determine whether it was, in fact, possible to identify activated DCs in the blood.

**Figure 2:**
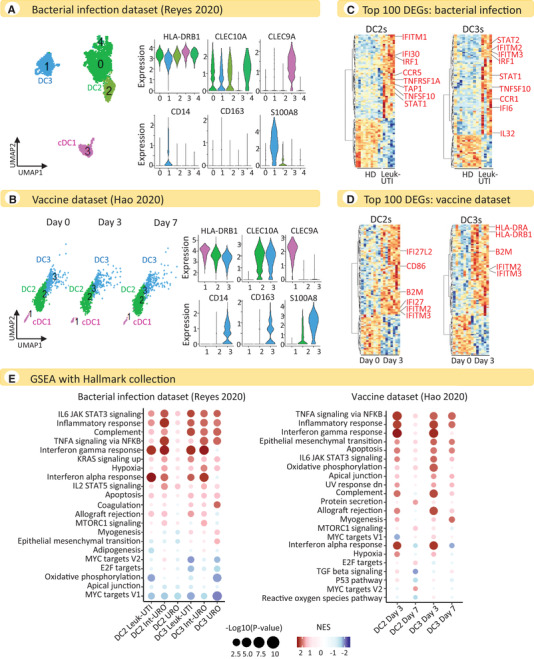
**Activation signature of cDCs during bacterial infection and adenovirus‐based vaccine administration**. (A) UMAP representations of cDC subpopulations and violin plots illustrating the expression levels of selected marker genes used for the manual annotation of cDC subsets in the bacterial infection dataset [29]. (B) UMAP representations of cDC subpopulations and violin plots illustrating the expression levels of selected marker genes used for the manual annotation of cDC subsets in the adenovirus‐based vaccine dataset [30]. (C) Heatmaps showing the top 100 DEGs comparing Leuk‐UTI patients with HDs in the dataset of bacterial infections, separately for DC2s and DC3s. Differential expression analysis was performed using pseudo‐bulk counts and, for each DC subset, only donors with at least 10 cells were retained (DC2s: *n* = 11 HDs, *n* = 5 Leuk‐UTI; DC3s: *n* = 10 HDs, *n* = 4 Leuk‐UTI). Selected upregulated genes are marked in red. Ribosomal protein (RP) genes were removed from the top 100 DEGs (see Supporting Information Table S4 for the full list of DEGs). (D) Heatmaps showing the top 100 DEGs comparing vaccinated donors at day 3 with unvaccinated donors in the vaccine dataset, separately for DC2s and DC3s. Differential expression analysis was performed using pseudo‐bulk counts and, for each DC subset, only donors with at least 10 cells were retained (DC2s: *n* = 8 unvaccinated, *n* = 8 vaccinated on day 3; DC3s: *n* = 7 unvaccinated, *n* = 7 vaccinated on day 3). Selected upregulated genes are marked in red. Ribosomal protein (RP) genes were removed from the top 100 DEGs (see Supporting Information Table S4 for the full list of DEGs). (E) GSEA of DEGs using the Hallmark collection: bacterial infection dataset (left panel) and vaccine dataset (right panel). For each dataset, the top 10 pathways in each cDC subset were selected and consolidated across all DC subsets in a single dot plot. NES, normalized enrichment score. Leuk‐UTI, urinary tract infection with leukocytosis. Int‐URO, intermediate urosepsis. URO, urosepsis.

We, therefore, investigated the transcriptional responses of circulating DC2 and DC3 subsets at single‐cell resolution under different clinical conditions. Two distinct publicly available datasets were analyzed: the dataset from Reyes *et al*. [29], containing scRNA‐seq data of PBMCs and enriched DCs obtained from patients with urinary tract bacterial infections of increasing severity (localized infection [Leuk‐UTI] or systemic infection with transient [Int‐URO] or persistent organ dysfunction [URO]), and the dataset from Hao *et al*. [30], containing CITE‐seq data of PBMCs obtained from healthy volunteers who received an adenovirus‐based vaccine. As already described, we performed dimensionality reduction and unsupervised clustering to identify cDC subpopulations. Our approach clearly identified cDC subsets in both datasets (Fig. 2A,B and Supplementary Fig. 7). We then determined DEGs in infected or vaccinated donors with respect to the corresponding HDs (Table S4) and performed GSEA.

The results are in stark contrast to those obtained from COVID‐19 patients. Indeed, circulating DC2s and DC3s in both datasets showed upregulation of not only IFN pathways (as in COVID‐19) but also inflammatory signatures and genes relevant for immune responses (differently from COVID‐19) (Fig. 2C,D,E and Supplementary Fig. 8,9). The most significantly upregulated genes included several encoding activation molecules, such as *CCR1*, *CCR5*, *TNFSF10* (*CD253/TRAIL*), *TNFRSF1A*, and *IL32* [31, 32, 33, 34, 35], as well as classical markers of DC maturation, such as *HLA‐DR*, *B2M*, *CD86*, and *TAP* (Fig. 2C,D and Supplementary Fig. 8A,B).

These findings were confirmed by pathway analysis, which showed a clear upregulation of activation pathways in DC2 and DC3 subsets in response to bacterial infection or vaccination, such as the inflammatory response pathway and the TNF‐α signaling pathway (Fig. 2E and Supplementary Fig. 9A,B). The leading edge genes driving the enrichment of the inflammatory response pathway in response to bacterial infections included several relevant for T‐cell activation (*IL1B, CCL5, TNFSF10, GPR183, CD69, SELL)* (Supplementary Fig. 9C), suggesting that cDC2s were undergoing conventional maturation. We found that the allograft rejection pathway in the Hallmark Gene Sets Collection, upregulated in both the bacterial infection and vaccine datasets, could be used as a proxy for the antigen‐presentation pathway, as many of the genes included in this gene set are involved in antigen presentation by DCs. Specifically, genes involved in the endogenous pathway of antigen presentation, including *HLA‐A*, *B2M*, *TAP1*, and *TAP2*, were all upregulated (Supplementary Fig. 10). Interestingly, we observed a stronger activation response for circulating cDC2s from patients with localized bacterial infections (Leuk‐UTI group) and transient organ dysfunction (Int‐URO group) than those with bacterial sepsis and persistent organ dysfunction (URO group) (Fig. 2E **, left panel**). This was expected, as sepsis induces functional impairment of myeloid cells.

### DC2s and DC3s respond similarly to SARS‐CoV‐2 infection and CD14^+^CD163^+^ DC3s accumulate in blood

**Figure 3:**
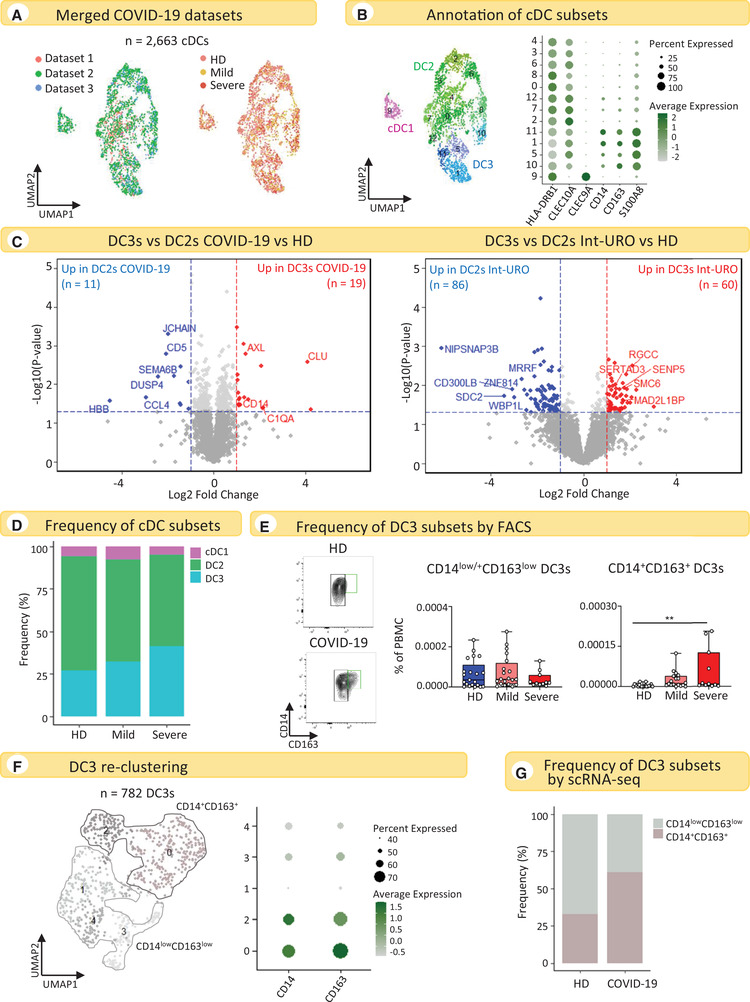
**DC2s and DC3s respond similarly to SARS‐CoV‐2 infection and CD14^+^CD163^+^ DC3s accumulate in COVID‐19 patients**. (A) UMAP representations of cDCs from three COVID‐19 datasets merged. Cells are colored according to the original dataset (left panel) and clinical condition (right panel). (B) UMAP representation of cDC subtypes and the corresponding dot plot illustrating the average expression of selected marker genes used for the manual annotation of cDC subtypes. (C) Volcano plots showing genes differentially induced in DC3s relative to DC2s in response to COVID‐19 (left panel: *n* = 7 HDs, *n* = 10 COVID‐19) and intermediate urosepsis (Int‐URO condition from the Reyes et al. dataset, right panel: *n* = 9 HDs, *n* = 4 Int‐URO). Genes with a *p*‐value < 0.05 and absolute log_2_ fold change > 1 were considered significant. Selected genes are highlighted (red: up in DC3s COVID‐19/Int‐URO, blue: up in DC2s COVID‐19/Int‐URO; see Supporting Information Table S5 for the full list of DEGs). (D) Bar plots showing the relative abundance of cDC populations in HDs and mild and severe COVID‐19 patients. (E) Contour plots showing CD14^+^CD163^+^ DC3s and CD14^low/+^CD163^low^ DC3s in a representative HD and COVID19 patient (left panel). Percentage of CD14^low/+^CD163^low^ DC3s and CD14^+^CD163^+^ DC3s among PBMCs from the whole blood of COVID‐19 patients (*n* = 22 mild and *n* = 10 severe) and HDs (*n* = 21). **p < 0.01 (right panel). Each dot represents an individual donor. (F) Re‐clustering of DC3s identified in Fig. 3B (left panel) and dot plot showing the average expression of CD14 and CD163 (right panel). (G) Bar plots showing the relative abundance of CD14^+^CD163^+^ DC3s and CD14^low^CD163^low^ DC3s in HDs and COVID‐19 patients.

Recent studies have shown potential functional differences between DC2s and DC3s [36] in inflammatory diseases, such as Systemic Lupus Erythematosus (SLE), in which type I IFNs play a major role [16]. We investigated the potential specific role of DC3s with respect to DC2s by determining the genes that are differentially induced/downmodulated by these two subpopulations in response to SARS‐CoV‐2 stimulation and compared them to bacterial infection. The resolution was increased by pooling cDCs from the three COVID‐19 datasets and performing Harmony integration [37], followed by graph‐based clustering. After integration, we obtained 2,663 cDCs (Fig. 3A) and clearly identified cDC1s, DC2s, and DC3s (Fig. 3B).

Only 30 genes (p‐value < 0.05 and absolute log_2_ fold change > 1) were differentially expressed in DC3s relative to DC2s in response to COVID‐19 infection, of which 19 were upregulated and 11 downregulated (Fig. 3C **, left panel** and Table S5). On the other hand, 146 genes (p‐value < 0.05 and absolute log_2_ fold change > 1) were identified as being differentially regulated in DC3s relative to DC2s in response to intermediate urosepsis (Int‐URO condition), of which 60 were upregulated and 86 downregulated (Fig. 3C **, right panel** and Table S5).

In conclusion, these findings suggest that DC2s and DC3s respond similarly to SARS‐CoV‐2 infection, whereas they show more diversified responses to bacterial infections.

Among the small number of genes differentially expressed between DC3s and DC2s in response to COVID‐19 were those encoding complement factors (*C1QA*) and, most importantly, anti‐apoptotic genes, such as *AXL* and *CLU*, which were among the most significantly upregulated (Fig. 3C **, left panel)**. This suggests that DC3s are less susceptible to apoptosis than DC2s and may explain why they tend to increase during SARS‐CoV‐2 infection (Fig. 1A). Comparison of these results with those obtained from bacterial infections showed genes associated with cell‐cycle progression and cell proliferation *(RGCC, SENP5, SMC6, SERTAD3, MAD2L1BP*) to be specifically upregulated in DC3s (Fig. 3C **, right panel**). Therefore, DC3s may proliferate during inflammatory responses or circulating DC3s may contain proliferating progenitors that expand the DC3 population during bacterial infections.

Coherent with these observations and the frequency of cDCs found in the blood of COVID‐19 patients (Fig. 1A), we also observed an alteration in the relative abundance of cDCs by scRNA‐seq. Specifically, DC3s showed higher frequencies in COVID‐19 patients with respect to HDs (Fig. 3D).

DC3s have been described as a heterogeneous population [16, 18], which was confirmed by our FACS analysis (Fig. 3E and Supplementary Fig. 1). CD1c^+^CD5^‐^ cells included CD14^low/+^CD163^low/+^ cells, for which the abundance differed between HDs and COVID‐19 patients. CD14^+^CD163^+^ cells were present in COVID‐19 patients but almost absent in HDs, who instead showed only CD14^low/+^CD163^low^ cells (Fig. 3E). Consistent with these findings, there was a significant increase in the frequency of CD14^+^CD163^+^ DC3s, but not of CD14^low/+^CD163^low^ DC3s, in the blood of COVID‐19 patients (Fig. 3E). We then investigated whether this heterogeneity was also measurable at the transcriptional level. Hence, we retained clusters annotated as DC3s (Fig. 3B) and performed a re‐clustering. We obtained a continuum of cells in which two subpopulations could be identified based on the expression of CD14 and CD163 (CD163^+^CD14^+^ and CD163^low^CD14^low^ cells) (Fig. 3F). Mirroring previous FACS analysis (Fig. 3E), we found an increase in the relative abundance of CD163^+^CD14^+^ DC3s in COVID‐19 patients relative to HDs (Fig. 3G).

### cDC2s skew towards inflammation and lose the Ag presenting function as disease severity increases

We investigated cDC2 gene expression profiles in COVID‐19 patients with severe versus mild disease to seek specific alterations in the innate immune signature between these two groups of patients and to link variations in the immune response to disease severity. As already described, we aggregated cell‐level counts into sample‐level pseudo‐bulk counts and identified DEGs between COVID‐19 patients with severe and mild disease (Table S6).

**Figure 4:**
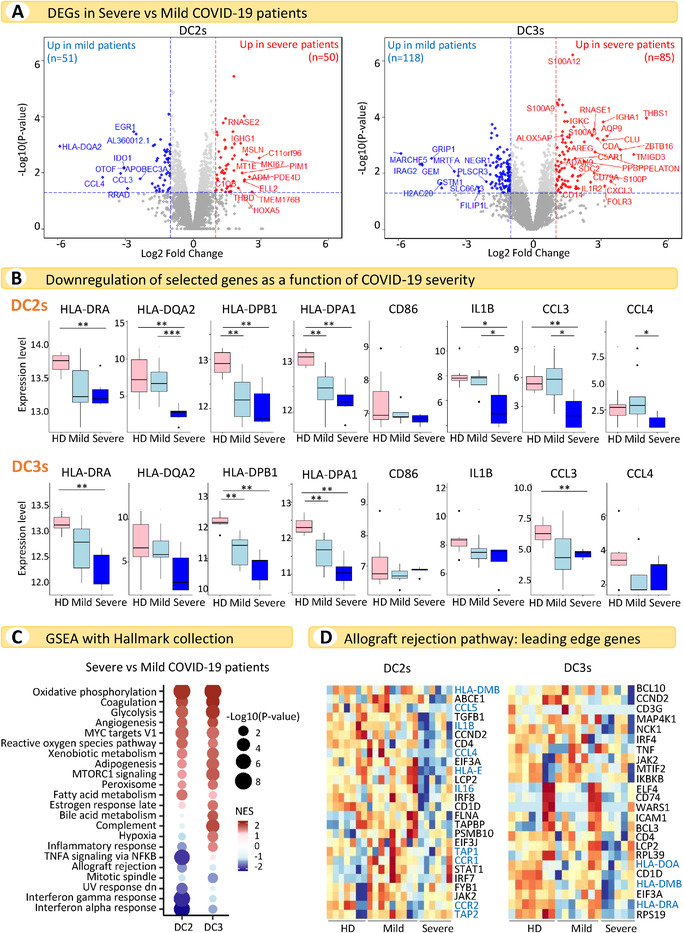
**cDC2s enhance inflammatory properties and lose their antigen presentation capacity in severe COVID‐19 patients**. (A) Volcano plots showing DEGs in COVID‐19 patients with severe versus mild disease in DC2s (left panel, *n* = 9 mild and *n* = 6 severe) and DC3s (right panel, *n* = 7 mild and *n* = 5 severe). Genes with a *p*‐value < 0.05 and absolute log_2_‐fold change > 1 were considered significant. Selected genes are highlighted (red: up in severe disease, blue: up in mild disease; see Supporting Information Table S6 for the full list of DEGs). (B) Boxplots showing expression levels of selected genes in DC2s and DC3s in HDs and patients with mild and severe disease (DC2s: *n* = 7 HDs, *n* = 9 mild, *n* = 6 severe; DC3s: *n* = 7 HDs, *n* = 7 mild, *n* = 5 severe). Data are represented as boxes and whiskers showing the median, interquartile range, and min to max. Individual points represent outliers. Statistical analyses were performed using the Wilcoxon rank sum test. *p < 0.05, **p < 0.01, ***p < 0.005. (C) GSEA of DEGs in patients with severe versus mild disease using the Hallmark collection. For each dataset, the top 15 pathways in each cDC subset were selected and consolidated across all DC subsets in a single dot plot. NES, normalized enrichment score. (D) Heatmaps showing GSEA leading edge genes of the allograft rejection pathway in DC2s and DC3s from HDs and COVID‐19 patients with mild and severe disease (DC2s: *n* = 7 HDs, *n* = 9 mild, *n* = 6 severe; DC3s: *n* = 7 HDs, *n* = 7 mild, *n* = 5 severe). Genes involved in relevant functions for DC‐mediated T‐cell activation are highlighted in blue.

We identified a large number of DEGs in both DC2s and DC3s (101 for DC2s and 203 for DC3s, p‐value < 0.05 and absolute log_2_ fold change > 1) between patients with severe and mild disease, indicating relevant differences in the transcriptional response of these two groups (Fig. 4A). Inflammatory genes not directly related to the activation of adaptive immunity, such as complement factors (*C1QB*) and complement receptors (*C5AR1*), those involved in the production of leukotrienes known to exacerbate respiratory syndromes (*ALOX5AP*) and the coagulation cascade (*THBS1, THBD*), those coding for factors involved in vasodilation (*ADM*), and other inflammatory genes, such as *CD14*, *S100A8/A9/A12* and *ADAM9*, were significantly upregulated in patients with severe versus mild disease in DC2s and/or DC3s (Fig. 4A). In addition, genes that negatively interfere with the maturation of DCs for T‐cell activation, such as *TMEM176B* [40] and *MT1E* [41], were upregulated in patients with severe versus those with mild disease (Fig. 4A). Moreover, *CLU* was among the significantly upregulated genes in DC3s of patients with severe disease, further supporting a potential role of anti‐apoptotic genes in DC3s during infections (Fig. 4A, right panel).

Strikingly, genes encoding MHCII molecules, the costimulatory molecule CD86, and cytokines, such as IL‐1β, CCL3, and CCL4, showed progressive downregulation in DC2s and/or DC3s from HDs to patients with mild disease and, finally, those with severe disease (Fig. 4B).

These observations were consolidated by pathway analysis, which showed clear upregulation of pathways involved in metabolism, coagulation, angiogenesis, and reactive oxygen species in patients with severe versus mild disease (Fig. 4C). Among the leading edge genes of the allograft rejection pathway, which was found to be downregulated in patients with severe versus mild disease, were many critical for DC‐mediated T‐cell activation, such as those coding for proteins involved in antigen presentation in both the MHCI and MHCII pathways (*TAP1, TAP2*, *HLA‐DMB*, *HLA‐DRA*) and genes encoding molecules relevant for T‐cell recruitment and activation (*IL16*, *IL1B*, *CCL4, CCL5, CCR1, CCR2*) (Fig. 4D). The specific downregulation of these genes in severely ill patients emphasizes the alteration of cDC functions in these individuals, which may be associated with worse disease progression.

### Direct activation of DCs by SARS‐CoV‐2

**Figure 5:**
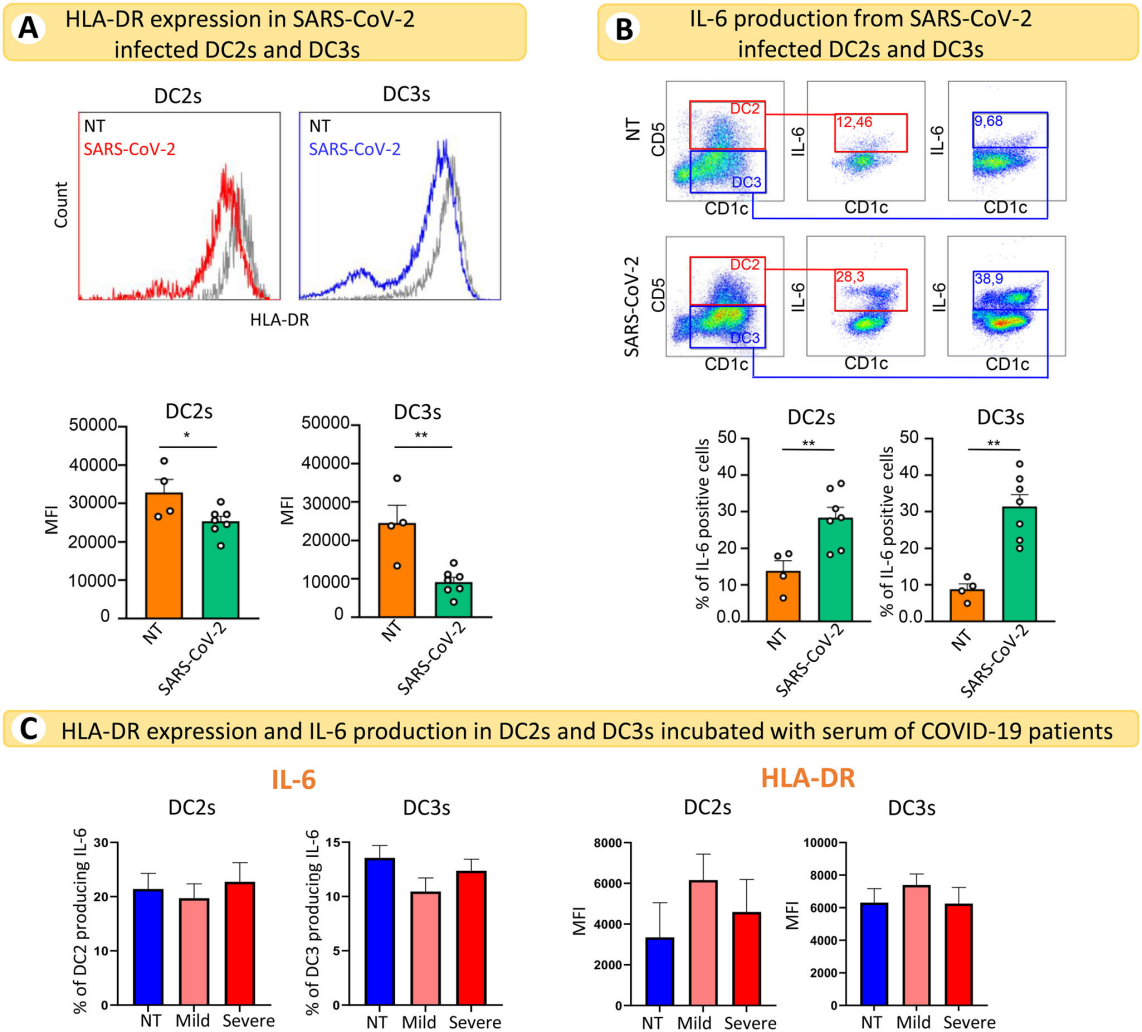
**SARS‐CoV‐2 directly induces downregulation of HLA‐DR and the production of IL‐6 in DC2s and DC3s**. (A, upper panel) Representative histograms showing HLA‐DR expression in cDC2s from HDs infected or not (NT) with 0.4 MOI of SARS‐CoV‐2 for 18 h. DC2s and DC3s were identified as CD5^+^CD1c^+^ and CD5^–^CD1c^+^, respectively, among CD11^+^LIN^–^ (CD88, CD89, CD3, and CD19) and FcεRIα^+^ cells. (A, lower panel) Quantitative analysis of the MFI of HLA‐DR in DC2s and DC3s. Statistical significance was determined using the unpaired Student's *t*‐test. *p < 0.05, **p < 0.01; *n* = 4 NT donors and *n* = 7 donors for SARS‐CoV‐2 infection. Each dot represents an individual donor. (B, upper panel) Representative dot plots showing the percentage of IL‐6‐producing DC2s and DC3s after viral infection, as described in (B). (B, lower panel) Quantitative analysis of the percentage of IL‐6‐producing cells. Statistical significance was determined using the unpaired Student's *t*‐test. **p < 0.01; *n* = 4 NT donors and *n* = 7 donors for SARS‐CoV‐2 infection. Each dot represents an individual donor. (C, left panel) Quantitative analysis of the percentage of IL‐6‐producing DC2s and DC3s (derived from 4 individual donors) after 18 h of incubation with sera from COVID‐19 patients with mild (*n* = 4) or severe (*n* = 4) disease. Each donor has been treated with sera from one HD, one severe and one mild COVID‐19 patient. NT: not treated. (C, right panel) Quantitative analysis of the MFI of HLA‐DR in DC2s and DC3s treated or not (NT) for 18 h with sera from COVID‐19 patients with mild (*n* = 4) or severe (*n* = 4) disease.

Finally, we investigated whether the direct interaction of cDC2s with the virus could induce a similar response to that observed at single‐cell resolution. We measured the response to the virus of cDC2s (CD1c^+^CD19^‐^ cells) freshly isolated from HDs using IL‐6 and MHCII as readouts. As predicted, we found that SARS‐CoV‐2 directly induced significant downregulation of MHCII surface expression and the upregulation of IL‐6 in both DC2s and DC3s (Fig. 5A,B). Conversely, the exposure of cDC2s to sera of patients with mild and severe disease, which contain inflammatory cytokines and other mediators, did not induce any modification in MHCII or IL‐6 expression (Fig. 5C).

## Discussion

In this study, we leveraged single‐cell technologies to thoroughly investigate the transcriptional signatures of cDCs during COVID‐19 pathogenesis in order to provide new information to understand the immune system's reaction to this specific infection.

We showed that, during SARS‐CoV‐2 infections, transcription signatures expressed by circulating cDC2s are characterized by the expression of ISGs and genes encoding factors of IL‐6‐induced pathways. Furthermore, by comparing the responses of severe and mild patients, we found a progressive downregulation, with the increasing of disease severity, of genes encoding signal 1 and signal 2 molecules associated with antigen presentation, and the upregulation of an inflammatory signature typical of innate immunity, mainly represented by complement and coagulation factors. Therefore, during COVID‐19, as disease severity increases, cDC2s progressively tend towards inflammatory activity and lose their antigen‐presenting function. This could explain the alteration of the T cell compartment seen in seriously ill patients.

The signatures we observed in COVID‐19 were typical of SARS‐CoV‐2 infections in hospitalized individuals. We analyzed datasets from patients with other types of infections, such as bacterial infections, and from vaccinated individuals. We found transcription signatures that reflected, as expected, the upregulation of DC antigen presenting functions. This was particularly evident in vaccinated individuals where a consistent upregulation of MHC and costimulatory molecules was observed. Therefore, SARS‐CoV‐2 can induce a kind of DC functional paralysis related to disease severity. It would be interesting to characterize the response of cDCs in SARS‐CoV‐2 infected pauci‐symptomatic patients to determine whether protective T cell responses are, in fact, associated with a mature DC phenotype.

While DC2 and DC3 subsets exhibited very similar responses in COVID‐19 patients, their responses were highly diversified in patients with bacterial infections. This finding may be explained, at least partially, by the differential expression of certain receptors, such as CD14, exclusively expressed by DC3s. CD14 is a component of the receptor complex of lipopolysaccharide (LPS), a major factor of the outer membrane of Gram‐negative bacteria, and contributes to LPS recognition and internalization of the receptor complex [38]. Thus, DC3s can respond more efficiently to Gram‐negative bacteria than DC2s. CD14 also has an important role as a chaperon for ligands of endosomal and cytosolic PRRs [38]. Therefore, the differences between DC2 and DC3 responses observed in Gram‐negative bacterial infections may also occur after Gram‐positive bacterial recognition. Further studies are needed to clarify potential differences between DC2 and DC3 functions during different viral infections.

Interestingly, some anti‐apoptotic genes, such as *AXL* and *CLU*, are differentially regulated between DC2s and DC3s during SARS‐CoV‐2 infections. This result, in combination with the increased frequencies observed in DC3s in COVID‐19 patients compared to HDs and the DC3 expansion shown in previous studies [16, 39], may explain why DC3s increase in number in acute and chronic inflammatory conditions.

The three main features we observed in cDC transcriptional responses (i) upregulation of ISGs and IL‐6 pathways, ii) progressive downregulation of genes encoding signal 1 and signal 2 molecules associated with antigen presentation, and iii) upregulation of an inflammatory signature) may be due to both the exposure to mediators released during SARS‐CoV‐2 infection or to the direct interaction of cDCs with the virus. Specifically, the first cDC characteristic observed in our study is compatible with both direct interaction with the virus and exposure to paracrine cytokines, such as IFNs and IL‐6 produced by bystander cells. The lack of expression of *IFN* and *IL6* genes in circulating DCs does not necessarily mean that cDCs cannot be a source of these cytokines, as their expression is acutely regulated and may be repressed in DCs early after activation. By contrast, the systematic downregulation of genes encoding MHCII molecules is more likely explained by a direct interaction of cDCs with the virus. According to this prediction and to the observation that the virus can activate monocyte‐derived DCs following abortive infection [42], we demonstrated that SARS‐CoV‐2 can directly induce significant downregulation of MHCII surface expression and the upregulation of IL‐6 in both DC2s and DC3s. On the other hand, these modifications are not achieved by exposure to COVID‐19 patients’ sera that contain inflammatory mediators. This suggests that at least part of the peculiar response of cDCs observed during SARS‐CoV‐2 infection may be directly mediated by the virus.

cDCs are likely to detect the virus through C‐type lectins, receptors that recognize N‐glycosylation on the Spike protein and identified as being responsible for myeloid‐cell activation [43]. C‐type lectins can bind to many different viruses. However, in addition to playing a positive role in antiviral responses by inducing the activation of DCs, they also contribute to viral spread through the migratory capacity of DCs [44, 45]. It would be informative to determine whether and where the activated DCs present in the circulation encountered the virus. In the case of cytomegalovirus infections in the mouse, it has been shown that infected DCs reach the lymph nodes and then exit them through high endothelial venules to reach the circulation [46]. Therefore, it is possible that DCs encounter the virus in the lungs and then reach the circulation, with migration favored by the strong inflammatory condition. Alternative possibilities are also plausible, such as the encounter of the virus by immature DCs directly in the blood or even in the bone marrow in severely ill patients.

In conclusion, SARS‐CoV‐2, like other viruses, can be directly detected by DCs and induces the downregulation of signals necessary for T‐cell activation, a phenomenon that is accentuated with disease severity. This allows the virus to evade control of the adaptive immune system, while the host attempts to counteract the viral infection through innate immunity. Understanding how DCs manage SARS‐CoV‐2 infection will help in identifying ad hoc interventions to achieve optimal adaptive responses, a prerequisite for a good prognosis [23, 47].

## Materials and methods

### Patients

COVID‐19 patients were enrolled in the STORM cohort (https://www.medicina.unimib.it/it/ricerca/covid‐19‐studi‐clinici). The local Ethical Committee approval was received for the study and the informed consent of all participating subjects was obtained.

### Flow cytometric analysis

PBMCs from COVID‐19 patients were extracted from peripheral blood by density gradient centrifugation using Ficoll (GE Healthcare). Cells were washed twice and stained for 30 min on ice using the following anti‐human antibodies (1:200, Becton Dickinson): anti‐FcεRIα PE‐Cy7, anti‐CD14 PE, anti‐CD1c APC‐Cy7, anti‐Clec9 (CD370) Alexa 647, anti‐CD5 BV786, anti‐CD3 BV605, anti‐CD19 BV605, anti‐CD88 BV605, anti‐CD89 BV605, anti‐CD11c BV480, anti‐CD163 BV421, and anti‐HLA‐DR BUV805. Cells were then washed and fixed using fixation buffer (Becton Dickinson) and the data acquired using a BD FACSsymphony instrument (Becton Dickinson). Analyses were performed using FlowJo X according to Cossarizza et al [48].

### cDC2 purification and activation

Human cDC2 cells were purified from PBMC extracts from the buffy coat of HDs (provided by the Niguarda Hospital blood bank) by Ficoll‐Paque density gradient centrifugation. Briefly, blood was stratified on Ficoll‐Paque PLUS (GE Healthcare) at a 3:4 ratio and centrifuged at 1500 rpm for 30 min without braking. PBMCs were washed twice, collected, and CD1c^+^ cells purified using MACS beads according to the manufacturer's instructions (Miltenyi Biotec). Cells were cultured in Roswell Park Memorial Institute (RPMI) 1640 medium (Euroclone) containing 10% heat‐inactivated fetal bovine serum (Euroclone), 100 IU of penicillin, streptomycin (100 μg/ml), and 2 mM l‐glutamine (Euroclone). cDC2s were infected with 0.4 MOI of SARS‐CoV‐2 for 18 h or treated with the serum of COVID‐19 patients (ratio serum/medium 1:1) and then collected and stained with anti‐FcεRIα PE‐Cy7, anti‐CD14 PE, anti‐CD1c APC‐Cy7, anti‐CD5 BV786, anti‐CD3 BV605, anti‐CD19 BV605, anti‐CD88 BV605, anti‐CD89 BV605, anti‐CD11c BV480, anti‐CD163 BV421, and anti‐HLA‐DR BUV805 (1:200, all from Becton Dickinson). Cells were then fixed and permeabilized using the Cytofix/Cytoperm™ Reagent Kit (Becton Dickinson) and stained with anti‐IL‐6 FITC antibody, according to the manufacturer's instructions. Samples were acquired using a BD FACSsymphony instrument (Becton Dickinson) and analyzed using Kaluza.

### Single‐cell RNA sequencing datasets analyzed in the study

Three different single‐cell datasets from COVID‐19 patients and healthy controls were analyzed in this study.

Dataset 1 was newly generated. Myeloid cells were sorted (CD11c^+^MHCII^+^) from three COVID‐19 patients (two with mild and one with severe disease, enrolled from the STORM cohort) and two HDs using a MACSQuant Tyto instrument (Miltenyi) (Supplementary Fig. 1B). After sorting, cell number and viability were evaluated using an automated cell counter. The viability of each sample was ≥75%. A Chromium Next GEM Chip G (10x Genomics) was loaded with 10,000 cells per sample. A Chromium controller (10× Genomics, Pleasanton, CA, USA) was used to generate single‐cell GEMs, according to the Chromium Next GEM Single Cell 5’ Library & Gel Bead Kit v1.1 protocol (PN‐1000165, 10× Genomics). Full‐length cDNA amplification and 5’ gene expression library construction were performed according to the manufacturer's instructions using a Veriti 96‐well Thermal Cycler (Thermo Fisher Scientific). Indexed libraries were sequenced using an Illumina Novaseq 6000 platform on a S2 flowcell, 150 bp PE (20,000 read pairs per cell). Reads from FASTQ files were aligned against the GRCh38 human reference genome and quantified using the Cell Ranger pipeline (10x Genomics) version 3.0 with default parameters.

Dataset 2 [22] was generated from a CITE‐seq experiment with PBMCs and enriched DCs from seven COVID‐19 patients (three with mild and four with severe disease) and five HDs. Count matrices were downloaded from the Gene Expression Omnibus (GEO) (GSE155673).

Dataset 3 [25] was generated from a scRNA‐seq experiment with PBMCs from 18 COVID‐19 patients (8 with mild and 10 with severe disease) that was integrated with publicly available 10x scRNA‐seq data of healthy controls from [29]. Seurat objects were downloaded from FASTGenomics (https://www.fastgenomics.org/). Only cells annotated as myeloid DCs by the authors were used in downstream analyses.

We analyzed two additional publicly available datasets to compare the transcriptional responses of cDC subsets between SARS‐CoV‐2 infection and other inflammatory conditions.

The Reyes *et al*. dataset [29] was generated from a scRNA‐seq experiment with PBMCs and enriched DCs obtained from patients with bacterial infections and healthy controls. Briefly, subjects were enrolled in two different cohorts. A primary cohort contained subjects that were classified into three clinical categories: Leuk‐UTI, Int‐URO, and URO. The Leuk‐UTI group refers to subjects with urinary‐tract infection (UTI) with leukocytosis (blood WBC ≥ 12,000 per mm^3^) but no organ dysfunction. The Int‐URO (intermediate urosepsis) group contains subjects with UTI with mild or transient organ dysfunction and the URO (urosepsis) group refers to subjects with UTI with clear or persistent organ dysfunction. Ten subjects were classified as Leuk‐UTI, seven as Int‐URO, and ten as URO. A second cohort was comprised of hospitalized subjects classified into three groups: subjects with bacteremia and sepsis not requiring intensive care unit (ICU) admission (Bac‐SEP group, four subjects), subjects with sepsis requiring ICU care (ICU‐SEP, eight subjects), and subjects in the ICU for conditions other than sepsis (ICU‐NoSEP, seven subjects). Data were downloaded from the Broad Institute Single Cell Portal (https://singlecell.broadinstitute.org/single_cell) (SCP548). We retained monocytes and DCs as annotated by the authors for downstream analysis.

The Hao *et al*. dataset [30] was generated from a CITE‐seq experiment with PBMCs from eight healthy volunteers enrolled in an adenovirus‐based HIV vaccine trial. For each subject, PBMCs were collected at three time points: immediately before (day 0) and 3 and 7 days following vaccine administration. Data were downloaded from https://atlas.fredhutch.org/nygc/multimodal‐pbmc/. We retained only cDCs as annotated by the authors for downstream analysis.

### Single‐cell data processing and analysis

Data processing and analysis for all single‐cell datasets was performed using the Seurat package (version 4.0.1) [30] in R (version 4.0.3).

First, filters were applied to remove low‐quality cells. These were based on the number of genes and UMIs detected in each cell and the percentage of reads mapping to mitochondrial genes (cells with <500 genes and >10% of reads mapping to mitochondrial RNA were removed). For COVID‐19 dataset 1, 16,325 genes and 15,400 cells were available before quality control (QC). After QC filtering, we retained 16,325 genes and 13,364 cells. For COVID‐19 dataset 2, 33,538 genes and 45,547 cells were available before QC. After QC filtering, we retained 33,538 genes and 33,430 cells. COVID‐19 dataset 3 was pre‐processed by the authors and 46,584 genes and 99,049 cells were available.

Counts were then normalized and log‐transformed using sctransform [49], while regressing out UMI counts and the percentage of mitochondrial counts.

PCA was performed to reduce dimensionality. Principal components (PCs) were fed to Harmony [37] for batch correction and/or the integration of datasets from both disease and healthy conditions. UMAP was used for 2D visualization. Clusters were identified using the shared nearest neighbor modularity optimization‐based clustering algorithm, followed by Louvain community detection. Cell type assignment was manually performed using marker genes, as detailed in the figures. cDCs were retained and again re‐clustered to identify subsets.

### Doublet identification

We observed a number of clusters likely comprised of primarily doublets, based on mixed lineage markers, when re‐clustering the cDCs in dataset 2. This was confirmed by two distinct doublet‐calling algorithms: the computeDoubletDensity method from scDblFinder package [50] and the cxds methods from the scds package [51].

### Pseudo‐bulk differential gene expression analysis

After the identification of cDC subsets, we aggregated cell‐level counts into sample‐level pseudo‐bulk counts. For each DC subset, only donors with at least 10 cells were retained. For the dataset from Reyes *et al*., only samples from the primary cohort were considered for differential analysis. Differential expression analysis was performed using the quasi‐likelihood framework of the edgeR package [52], using each donor as the unit of independent replication.

### Gene set enrichment analysis

Pre‐ranked GSEA [53] was performed on differentially expressed genes (DEGs) using the fgsea package [54]. The Hallmark Collection (MSigDB, Broad Institute) and the Blood Transcription Modules (BTM) [26] were used. BTM families analyzed in this study are reported in Supporting Information Table 3.

### Integration between COVID‐19 datasets

cDCs identified in the three COVID‐19 datasets were pooled, integrated using the Harmony algorithm [37], and further subclustered using the shared nearest neighbor modularity optimization‐based clustering algorithm, followed by Louvain community detection to identify cDC1s, DC2s, and DC3s. For dataset 3, only COVID‐19 samples were retained for the integration.

### Data and code availability

For COVID‐19 dataset 1, data supporting the findings of this study are available in GEO at https://www.ncbi.nlm.nih.gov/geo/, reference number GSE168388. For COVID‐19 dataset 2 [22], data supporting the findings of this study are available in GEO at https://www.ncbi.nlm.nih.gov/geo/, reference number GSE155673. For COVID‐19 dataset 3 [25], data supporting the findings of this study are available at FASTGenomics (https://www.fastgenomics.org/). For the bacterial infection dataset [29], data supporting the findings of this study are available at the Broad Institute Single Cell Portal (https://singlecell.broadinstitute.org/single_cell), reference number SCP548. For the vaccine dataset [30], data supporting the findings of this study are available at https://atlas.fredhutch.org/nygc/multimodal‐pbmc/. The analysis code is available at https://github.com/giuliaprotti/DC_COVID19.

## Author contributions

F.G. and L.M. were associated with study conceptualization. L.M., R.S., G.P., V.R., A.R.P., L.S., V.B., C.S., M.C.C., and S.A. were associated with methodology. L.M., G.P., R.S., F.A.F., M.V., F.M., S.A., S.C., M.D.A., L.B., A.B., L.N., and N.T. were associated with the investigations. F.G., G.P., R.S., and L.M. were associated with visualization. F.G. acquired the funding and supervised the study. F.G. and G.P. wrote the original draft of the manuscript. F.G., G.P., L.M., and R.S. reviewed and edited the final manuscript.

## Conflict of interest

R.S. is currently an employee of GlaxoSmithKline. The other authors declare that they have no competing interests.

### Peer review

The peer review history for this article is available at https://publons.com/publon/10.1002/eji.202149298

**Abbreviations**
ARDS: acute respiratory distress syndrome
BTM: blood transcription module
cDC: conventional DC
DIC: diffuse intravascular coagulation
GSEA: gene set enrichment analysis
HD: healthy donor
ISG: IFN‐stimulated gene
PBMC: peripheral blood mononuclear cell
PRR: pattern recognition receptor
ScRNA‐seq: single‐cell RNA sequencing
UMAP: uniform manifold approximation and projection

## Supporting information



Supporting Information

Table S1

Table S2

Table S3

Table S4

Table S5

Table S6

## References

[1] Silvin, A. , Chapuis, N. , Dunsmore, G. , Goubet, A. G. , Dubuisson, A. , Derosa, L. , Almire, C. et al., Elevated Calprotectin and abnormal myeloid cell subsets discriminate severe from mild COVID‐19. Cell 2020. 182: 1401–1418. 32810439 10.1016/j.cell.2020.08.002 PMC7405878

[2] Lucas, C. , Wong, P. , Klein, J. , Castro, T. B. R. , Silva, J. , Sundaram, M. , Ellingson, M. K. et al., Longitudinal analyses reveal immunological misfiring in severe COVID‐19. Nature 2020. 584: 463–469. 32717743 10.1038/s41586-020-2588-y PMC7477538

[3] Mangalmurti, N. and Hunter, C. A. , Cytokine storms: understanding COVID‐19. Immunity 2020. 53: 19–25. 32610079 10.1016/j.immuni.2020.06.017 PMC7321048

[4] Merrill, J. T. , Erkan, D. , Winakur, J. and James, J. A. , Emerging evidence of a COVID‐19 thrombotic syndrome has treatment implications. Nat. Rev. Rheumatol. 2020. 16: 581–589. 32733003 10.1038/s41584-020-0474-5 PMC7391481

[5] Wu, Z. and McGoogan, J. M. , Characteristics of and important lessons from the coronavirus disease 2019 (COVID‐19) outbreak in China: summary of a report of 72314 cases from the Chinese center for disease control and prevention. JAMA ‐ J. Am. Med. Assoc. 2020. 323: 1239–1242. 10.1001/jama.2020.2648 32091533

[6] Laing, A. G. , Lorenc, A. , del Molino del Barrio, I. , Das, A. , Fish, M. , Monin, L. , Muñoz‐Ruiz, M. et al., A dynamic COVID‐19 immune signature includes associations with poor prognosis. Nat. Med. 2020. 26: 1623–1635. 32807934 10.1038/s41591-020-1038-6

[7] Wauters, E. , Van Mol, P. , Garg, A. D. , Jansen, S. , Van Herck, Y. , Vanderbeke, L. , Bassez, A. et al., Discriminating mild from critical COVID‐19 by innate and adaptive immune single‐cell profiling of bronchoalveolar lavages. Cell Res. 2021. 31: 272–290. 33473155 10.1038/s41422-020-00455-9 PMC8027624

[8] Cabeza‐Cabrerizo, M. , Cardoso, A. , Minutti, C. M. , Pereira da Costa, M. and Reis e Sousa, C. , Dendritic cells revisited. Annu. Rev. Immunol. 2021. 39: 131–166. 33481643 10.1146/annurev-immunol-061020-053707

[9] Eisenbarth, S. C. , Dendritic cell subsets in T cell programming: location dictates function. Nat. Rev. Immunol. 2019. 19: 89–103. 30464294 10.1038/s41577-018-0088-1 PMC7755085

[10] See, P. , Dutertre, C. A. , Chen, J. , Günther, P. , McGovern, N. , Irac, S. E. , Gunawan, M. et al., Mapping the human DC lineage through the integration of high‐dimensional techniques. Science (80‐.) 2017. 356: eaag3009. 10.1126/science.aag3009 PMC7611082 28473638

[11] Anderson, D. A. , Dutertre, C. A. , Ginhoux, F. and Murphy, K. M. , Genetic models of human and mouse dendritic cell development and function. Nat. Rev. Immunol. 2020. 21: 101–115. 32908299 10.1038/s41577-020-00413-x PMC10955724

[12] Guilliams, M. , Dutertre, C. A. , Scott, C. L. , McGovern, N. , Sichien, D. , Chakarov, S. , Van Gassen, S. et al., Unsupervised high‐dimensional analysis aligns dendritic cells across tissues and species. Immunity 2016. 45: 669–684. 27637149 10.1016/j.immuni.2016.08.015 PMC5040826

[13] Canton, J. , Blees, H. , Henry, C. M. , Buck, M. D. , Schulz, O. , Rogers, N. C. , Childs, E. et al., The receptor DNGR‐1 signals for phagosomal rupture to promote cross‐presentation of dead‐cell‐associated antigens. Nat. Immunol. 2021. 22: 140–153. 33349708 10.1038/s41590-020-00824-x PMC7116638

[14] Jongbloed, S. L. , Kassianos, A. J. , McDonald, K. J. , Clark, G. J. , Ju, X. , Angel, C. E. , Chen, C. J. J. et al., Human CD141+ (BDCA‐3)+ dendritic cells (DCs) represent a unique myeloid DC subset that cross‐presents necrotic cell antigens. J. Exp. Med. 2010. 207: 1247–1260. 20479116 10.1084/jem.20092140 PMC2882828

[15] Nizzoli, G. , Krietsch, J. , Weick, A. , Steinfelder, S. , Facciotti, F. , Gruarin, P. , Bianco, A. et al., Human CD1c+ dendritic cells secrete high levels of IL‐12 and potently prime cytotoxic T‐cell responses. Blood 2013. 122: 932–942. 23794066 10.1182/blood-2013-04-495424

[16] Dutertre, C. A. , Becht, E. , Irac, S. E. , Khalilnezhad, A. , Narang, V. , Khalilnezhad, S. , Ng, P. Y. et al., Single‐cell analysis of human mononuclear phagocytes reveals subset‐defining markers and identifies circulating inflammatory dendritic cells. Immunity 2019. 51: 573–589.e8. Available at:31474513 10.1016/j.immuni.2019.08.008

[17] Villani, A. C. , Satija, R. , Reynolds, G. , Sarkizova, S. , Shekhar, K. , Fletcher, J. , Griesbeck, M. et al., Single‐cell RNA‐seq reveals new types of human blood dendritic cells, monocytes, and progenitors. Science (80‐.) 2017. 356. 10.1126/science.aah4573. PMC5775029 28428369

[18] Cytlak, U. , Resteu, A. , Pagan, S. , Green, K. , Milne, P. , Maisuria, S. , McDonald, D. et al., Differential IRF8 transcription factor requirement defines two pathways of dendritic cell development in humans. Immunity. 2020. 53: 353–370.e8. 32735845 10.1016/j.immuni.2020.07.003 PMC7447982

[19] Sánchez‐Cerrillo, I. , Landete, P. , Aldave, B. , Sánchez‐Alonso, S. , Sánchez‐Azofra, A. , Marcos‐Jiménez, A. , Ávalos, E. et al., COVID‐19 severity associates with pulmonary redistribution of CD1c+ DCs and inflammatory transitional and nonclassical monocytes. J. Clin. Invest. 2020. 130: 6290–6300. 32784290 10.1172/JCI140335 PMC7685723

[20] Kvedaraite, E. , Hertwig, L. , Sinha, I. , Ponzetta, A. , Hed Myrberg, I. , Lourda, M. , Dzidic, M. et al., Major alterations in the mononuclear phagocyte landscape associated with COVID‐19 severity. Proc. Natl. Acad. Sci. 2021. 118: e2018587118 33479167 10.1073/pnas.2018587118 PMC8017719

[21] Zhou, R. , To, K. K. W. , Wong, Y. C. , Liu, L. , Zhou, B. , Li, X. , Huang, H. et al., Acute SARS‐CoV‐2 infection impairs dendritic cell and T cell responses. Immunity 2020. 53: 864–877.e5. 32791036 10.1016/j.immuni.2020.07.026 PMC7402670

[22] Arunachalam, P. S. , Wimmers, F. , Mok, C. K. P. , Perera, R. A. P. M. , Scott, M. , Hagan, T. , Sigal, N. et al., Systems biological assessment of immunity to mild versus severe COVID‐19 infection in humans. Science (80‐.) 2020. 369: 1210–1220. 10.1126/science.abc6261 PMC7665312 32788292

[23] Sette, A. and Crotty, S. , Adaptive immunity to SARS‐CoV‐2 and COVID‐19. Cell 2021. 184: 861–880. 33497610 10.1016/j.cell.2021.01.007 PMC7803150

[24] Marongiu, L. , Protti, G. and Granucci, F. , Maturation signatures of conventional dendritic cell subtypes in COVID‐19 suggest direct viral sensing. Gene Expression Omnibus (GEO) 2021. 10.1002/eji.202149298 PMC8420462 34333764

[25] Schulte‐Schrepping, J. , Reusch, N. , Paclik, D. , Baßler, K. , Schlickeiser, S. , Zhang, B. , Krämer, B. et al., Severe COVID‐19 is marked by a dysregulated myeloid cell compartment. Cell 2020. 182: 1419–1440.e23. 32810438 10.1016/j.cell.2020.08.001 PMC7405822

[26] Li, S. , Rouphael, N. , Duraisingham, S. , Romero‐Steiner, S. , Presnell, S. , Davis, C. , Schmidt, D. S. et al., Molecular signatures of antibody responses derived from a systems biology study of five human vaccines. Nat. Immunol. 2014. 15: 195–204. 24336226 10.1038/ni.2789 PMC3946932

[27] Pullamsetti, S. S. , Seeger, W. and Savai, R. , Classical IL‐6 signaling: A promising therapeutic target for pulmonary arterial hypertension. J. Clin. Invest. 2018. 128: 1720–1723. 29629898 10.1172/JCI120415 PMC5919797

[28] De Biasi, S. , Meschiari, M. , Gibellini, L. , Bellinazzi, C. , Borella, R. , Fidanza, L. , Gozzi, L. et al., Marked T cell activation, senescence, exhaustion and skewing towards TH17 in patients with COVID‐19 pneumonia. Nat. Commun. 2020. 11: 3434. 32632085 10.1038/s41467-020-17292-4 PMC7338513

[29] Reyes, M. , Filbin, M. R. , Bhattacharyya, R. P. , Billman, K. , Eisenhaure, T. , Hung, D. T. , Levy, B. D. et al., An immune‐cell signature of bacterial sepsis. Nat. Med. 2020. 26: 333–340. 32066974 10.1038/s41591-020-0752-4 PMC7235950

[30] Hao, Y. , Hao, S. , Andersen‐Nissen, E. , Mauck, W. M. , Zheng, S. , Butler, A. , Lee, M. J. et al., Integrated analysis of multimodal single‐cell data. bioRxiv 2020. 10.1101/2020.10.12.335331. bioRxiv PMC8238499 34062119

[31] Scholz, F. , Grau, M. , Menzel, L. , Graband, A. , Zapukhlyak, M. , Leutz, A. , Janz, M. et al., The transcription factor C/EBPβ orchestrates dendritic cell maturation and functionality under homeostatic and malignant conditions. Proc. Natl. Acad. Sci. U. S. A. 2020. 117: 26328–26339. 33020261 10.1073/pnas.2008883117 PMC7584915

[32] Lee, B. , Sharron, M. , Montaner, L. J. , Weissman, D. and Doms, R. W. , Quantification of CD4, CCR5, and CXCR4 levels on lymphocyte subsets, dendritic cells, and differentially conditioned monocyte‐derived macrophages. Proc. Natl. Acad. Sci. U. S. A. 1999. 96: 5215–5220. 10220446 10.1073/pnas.96.9.5215 PMC21844

[33] Fanger, N. A. , Maliszewski, C. R. , Schooley, K. and Griffith, T. S. , Human dendritic cells mediate cellular apoptosis via tumor necrosis factor‐related apoptosis‐inducing ligand (TRAIL). J. Exp. Med. 1999. 190: 1155–1164. 10523613 10.1084/jem.190.8.1155 PMC2195665

[34] Maney, N. J. , Reynolds, G. , Krippner‐Heidenreich, A. and Hilkens, C. M. U. , Dendritic cell maturation and survival are differentially regulated by TNFR1 and TNFR2. J. Immunol. 2014. 193: 4914–4923. 25288570 10.4049/jimmunol.1302929 PMC4896387

[35] Ribeiro‐Dias, F. , Saar Gomes, R. , de Lima Silva, L. L. , dos Santos, J. C. and Joosten, L. A. B. , Interleukin 32: a novel player in the control of infectious diseases. J. Leukoc. Biol. 2017. 101: 39–52. 27793959 10.1189/jlb.4RU0416-175RR

[36] Bourdely, P. , Anselmi, G. , Vaivode, K. , Ramos, R. N. , Missolo‐Koussou, Y. , Hidalgo, S. , Tosselo, J. et al., Transcriptional and functional analysis of CD1c+ human dendritic cells identifies a CD163+ subset priming CD8+CD103+ T cells. Immunity 2020. 53: 335–352.e8. 32610077 10.1016/j.immuni.2020.06.002 PMC7445430

[37] Korsunsky, I. , Millard, N. , Fan, J. , Slowikowski, K. , Zhang, F. , Wei, K. , Baglaenko, Y. et al., Fast, sensitive and accurate integration of single‐cell data with Harmony. Nat. Methods. 2019. 16: 1289–1296. 31740819 10.1038/s41592-019-0619-0 PMC6884693

[38] Zanoni, I. and Granucci, F. , Role of CD14 in host protection against infections and in metabolism regulation. Front. Cell. Infect. Microbiol. 2013. 4. 10.3389/fcimb.2013.00032. PMC3721004 23898465

[39] Bakdash, G. , Buschow, S. I. , Gorris, M. A. J. , Halilovic, A. , Hato, S. V. , Sköld, A. E. , Schreibelt, G. et al., Expansion of a BDCA1+ CD14+ myeloid cell population in melanoma patients may attenuate the efficacy of dendritic cell vaccines. Cancer Res. 2016. 76. 10.1158/0008-5472.CAN-15-1695. 27325645

[40] Segovia, M. , Louvet, C. , Charnet, P. , Savina, A. , Tilly, G. , Gautreau, L. , Carretero‐Iglesia, L. et al., Autologous dendritic cells prolong allograft survival through Tmem176b‐dependent antigen cross‐presentation. Am. J. Transplant. 2014. 14: 1021–1031. 24731243 10.1111/ajt.12708 PMC4629416

[41] García‐González, P. A. , Schinnerling, K. , Sepúlveda‐Gutiérrez, A. , Maggi, J. , Mehdi, A. M. , Nel, H. J. , Pesce, B. et al., Dexamethasone and monophosphoryl lipid a induce a distinctive profile on monocyte‐derived dendritic cells through transcriptional modulation of genes associated with essential processes of the immune response. Front. Immunol. 2017. 8. 10.3389/fimmu.2017.01350. PMC5660598 29109727

[42] Zheng, J. , Wang, Y. , Li, K. , Meyerholz, D. K. , Allamargot, C. and Perlman, S. , Severe acute respiratory syndrome coronavirus 2–induced immune activation and death of monocyte‐derived human macrophages and dendritic cells. J. Infect. Dis. 2020. 223: 785–795. 10.1093/infdis/jiaa753 PMC7799009 33277988

[43] Lu, Q. , Liu, J. , Zhao, S. , Gomez Castro, M. F. , Laurent‐Rolle, M. , Dong, J. , Ran, X. et al., SARS‐CoV‐2 exacerbates proinflammatory responses in myeloid cells through C‐type lectin receptors and Tweety family member 2. Immunity 2021: 1–16. Available at:34048708 10.1016/j.immuni.2021.05.006 PMC8106883

[44] Lambert, A. A. , Gilbert, C. , Richard, M. , Beaulieu, A. D. and Tremblay, M. J. , The C‐type lectin surface receptor DCIR acts as a new attachment factor for HIV‐1 in dendritic cells and contributes to trans‐ and cis‐infection pathways. Blood. 2008. 112: 1299–1307. 18541725 10.1182/blood-2008-01-136473 PMC2515113

[45] Simmons, G. , Reeves, J. D. , Grogan, C. C. , Vandenberghe, L. H. , Baribaud, F. , Whitbeck, J. C. , Burke, E. et al., DC‐SIGN and DC‐SIGNR bind Ebola glycoproteins and enhance infection of macrophages and endothelial cells. Virology 2003. 305: 115–123. 12504546 10.1006/viro.2002.1730

[46] Farrell, H. E. , Bruce, K. , Lawler, C. , Oliveira, M. , Cardin, R. , Davis‐Poynter, N. and Stevenson, P. G. , Murine cytomegalovirus spreads by dendritic cell recirculation. MBio 2017. 8: 1–13. 10.1128/mBio.01264-17 PMC5626969 28974616

[47] Tan, A. T. , Linster, M. , Tan, C. W. , Le Bert, N. , Chia, W. N. , Kunasegaran, K. , Zhuang, Y. et al., Early induction of functional SARS‐CoV‐2‐specific T cells associates with rapid viral clearance and mild disease in COVID‐19 patients. Cell Rep. 2021. 34: 108728. 33516277 10.1016/j.celrep.2021.108728 PMC7826084

[48] Cossarizza, A. , Chang, H. D. , Radbruch, A. , Acs, A. , Adam, D. , Adam‐Klages, S. , Agace, W. W. et al., Guidelines for the use of flow cytometry and cell sorting in immunological studies (second edition). Eur. J. Immunol. 2019. 49: 1457–1973. 31633216 10.1002/eji.201970107 PMC7350392

[49] Hafemeister, C. and Satija, R. , Normalization and variance stabilization of single‐cell RNA‐seq data using regularized negative binomial regression. Genome Biol. 2019. 20. 10.1186/s13059-019-1874-1. PMC6927181 31870423

[50] Germain, P. , scDblFinder. R package version 1.6.0, https://github.com/plger/scDblFinder. 2021.

[51] Bais, A. S. , Kostka, D. , Scds: Computational annotation of doublets in single‐cell RNA sequencing data. Bioinformatics. 2020. 36: 1150–1158. 31501871 10.1093/bioinformatics/btz698 PMC7703774

[52] Robinson, M. D. , McCarthy, D. J. and Smyth, G. K. , edgeR: A Bioconductor package for differential expression analysis of digital gene expression data. Bioinformatics 2009. 26: 139–140. 19910308 10.1093/bioinformatics/btp616 PMC2796818

[53] Subramanian, A. , Tamayo, P. , Mootha, V. K. , Mukherjee, S. , Ebert, B. L. , Gillette, M. A. , Paulovich, A. et al., Gene set enrichment analysis: A knowledge‐based approach for interpreting genome‐wide expression profiles. Proc. Natl. Acad. Sci. U. S. A. 2005. 102: 15545–15550. 16199517 10.1073/pnas.0506580102 PMC1239896

[54] Sergushichev, A. A. , An algorithm for fast preranked gene set enrichment analysis using cumulative statistic calculation. bioRxiv 2016.

