# Supplementary material for: Maturation signatures of conventional dendritic cell subtypes in COVID‐19 suggest direct viral sensing
Source: Eur J Immunol. 2021 Oct 1;52(1):109–22. doi: 10.1002/eji.202149298 (PMC8420462; doi:10.1002/eji.202149298)
Supplement: Supplementary file 1 — Supporting Information [file EJI-52-109-s004.pdf]

Supplementary Figures and Tables

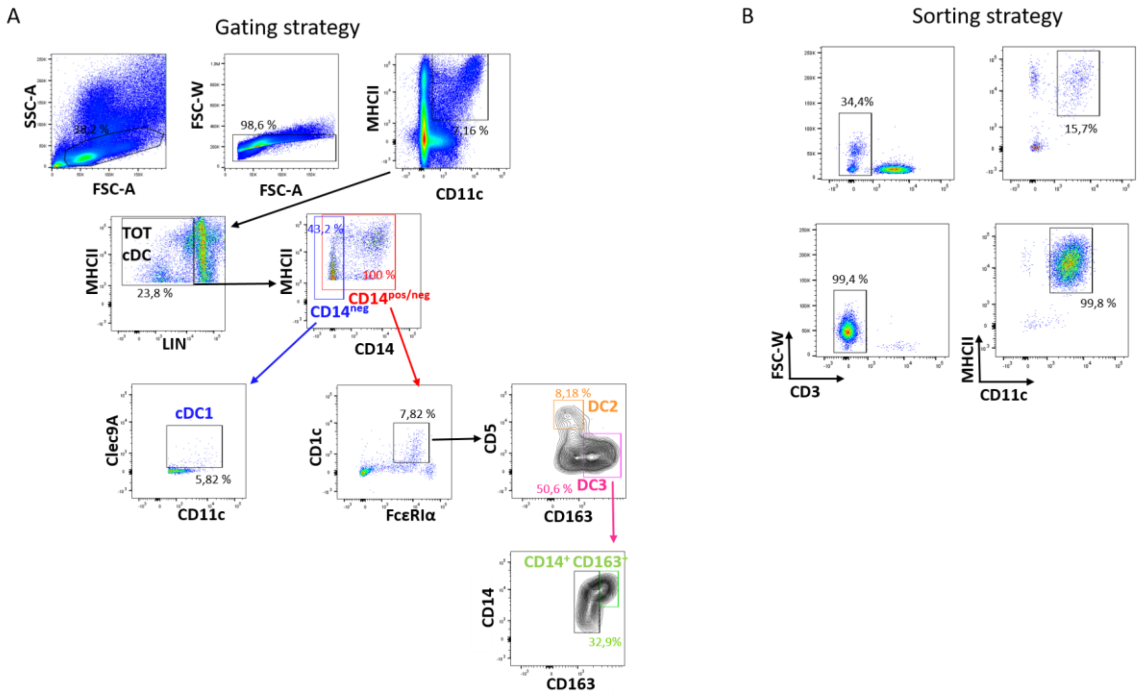

Supplementary Figure 1.

(A) Gating strategy used to identify DCs subsets from PBMCs. Total DCs (cDCs TOT) were detected among the CD11c$^+$MHCII$^+$ and LIN$^-$ (CD88, CD89, CD3 and CD19) population. cDC1s were identified as CLEC9A$^+$ from the CD14$^-$ fraction of total DCs. cDC2s (FcεRIα$^+$ CD1c$^+$) include CD14$^+$ and CD14$^-$ cells. DC2s and DC3s were identified as CD5$^+$CD163$^-$ and CD5$^-$CD163$^+$ respectively. Inflammatory DC3s were recognized as CD14$^+$CD163$^+$ cells. (B) Sorting strategy used to purify MHCII$^+$CD11c$^+$ cells; the upper panels show the pre-sorting population, the lower panels show the post-sorting population.

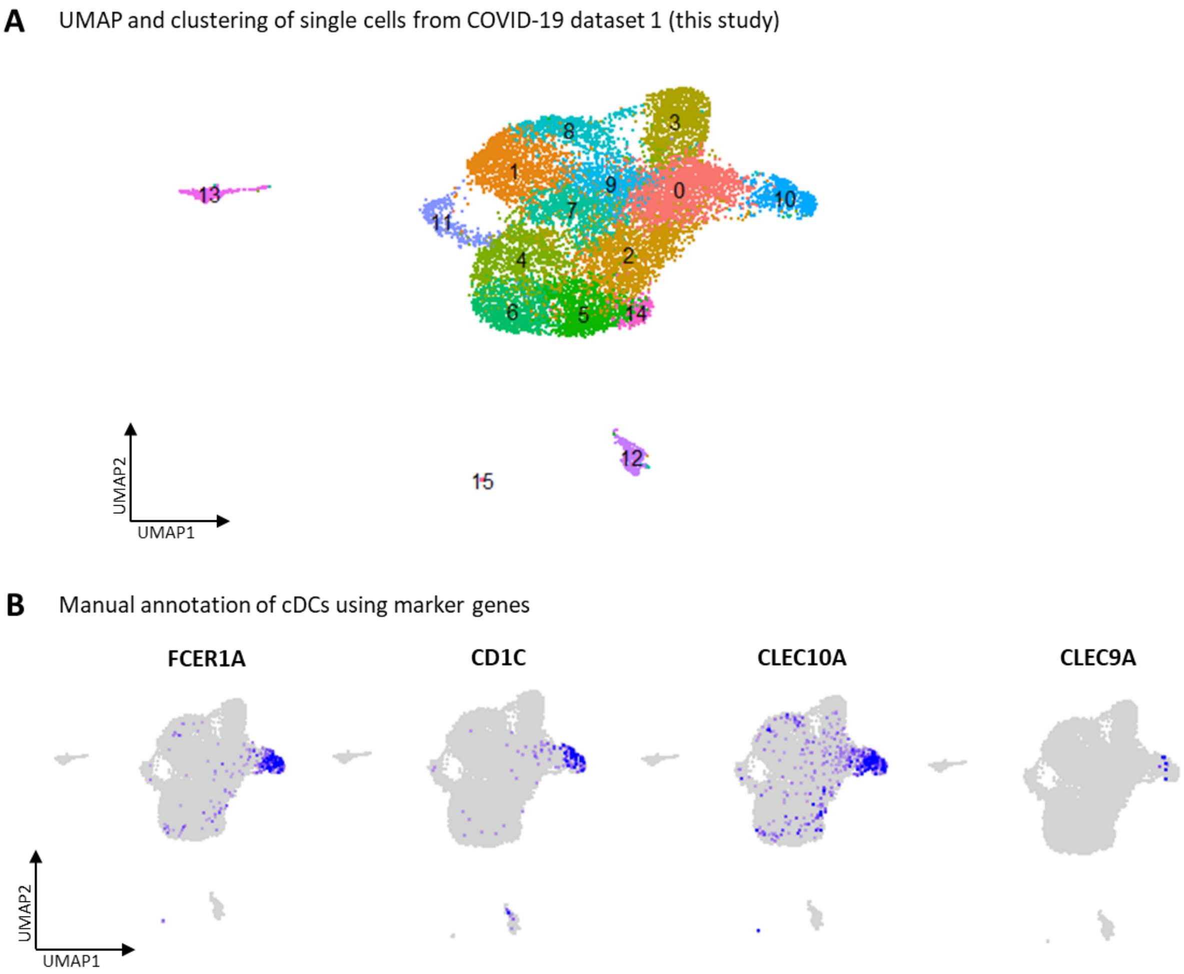

Supplementary Figure 2.

(A) UMAP and clustering of single cells from COVID-19 dataset 1 (this study). (B) Feature plots showing the expression levels of selected marker genes (FCER1A, CD1C, CLEC10A, CLEC9A) used to identify cDCs. Cluster 10, corresponding to cDCs, was re-clustered in a final iteration to clearly delineate cDC subsets as shown in Figure 1B.

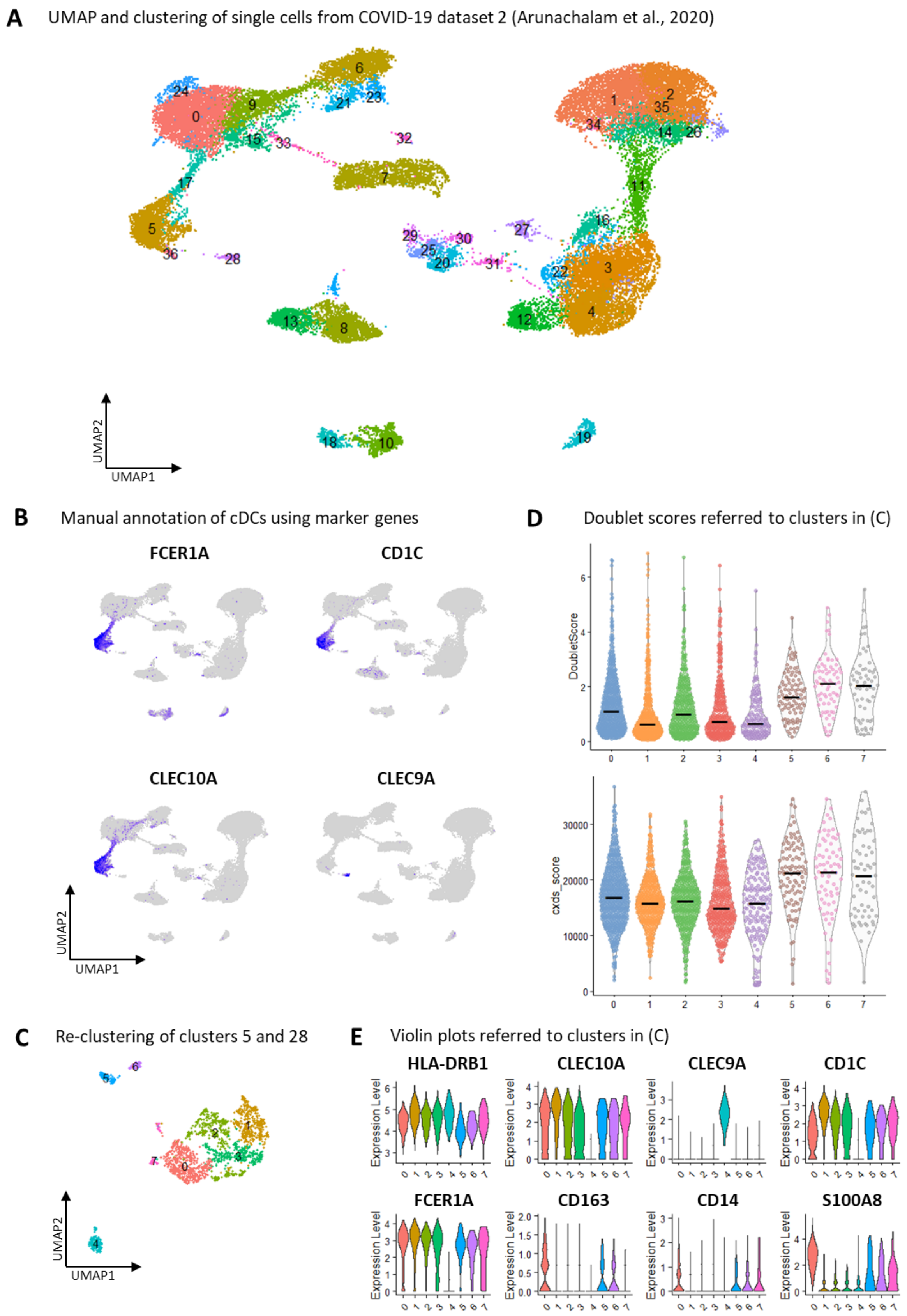

Supplementary Figure 3.

(A) UMAP and clustering of single cells from COVID-19 dataset 2 (Arunachalam et al., 2020).

(B) Feature plots showing the expression levels of selected marker genes (FCER1A, CD1C,

CLEC10A, CLEC9A) used to identify cDCs. (C) Re-clustering of clusters 5 and 28 corresponding to cDCs. (D) Violin plots showing doublet scores assigned to clusters in (C) using two different methods for *in silico* doublet identification: the computeDoubletDensity method from scDblFinder package (top panel) and the cxds methods from scds package (bottom panel). Black horizontal line is the median. Clusters 5, 6 and 7 were identified as doublets and removed. (E) Violin plots referred to clusters in (C) showing expression levels of selected marker genes. Clusters 0, 1, 2, 3 and 4 were re-clustered in a final iteration to clearly delineate cDC subsets as shown in Figure 1B.

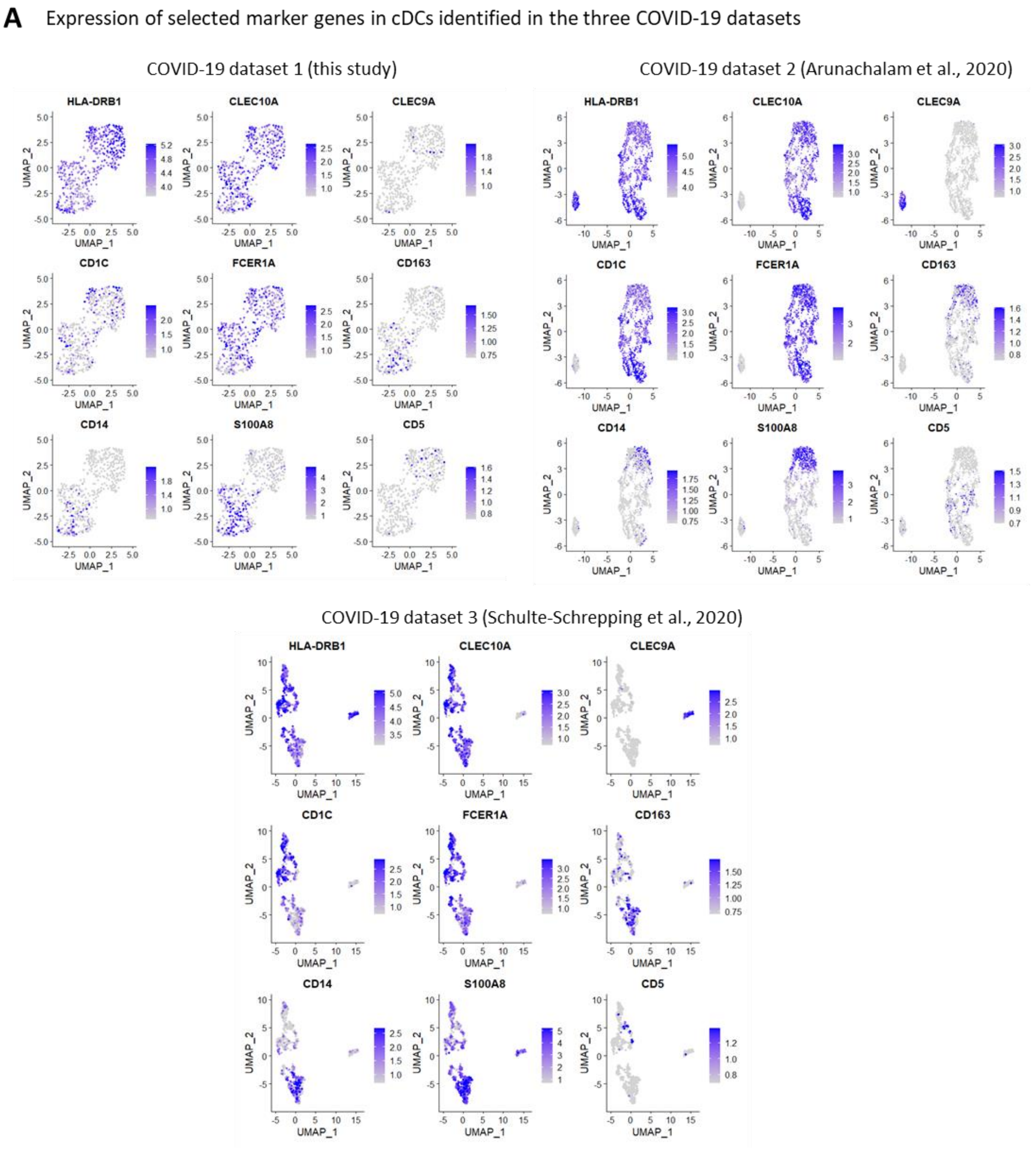

Supplementary Figure 4.

(A) Feature plots referred to Figure 1B showing expression levels of selected marker genes in cDCs identified in the three COVID-19 datasets analysed.

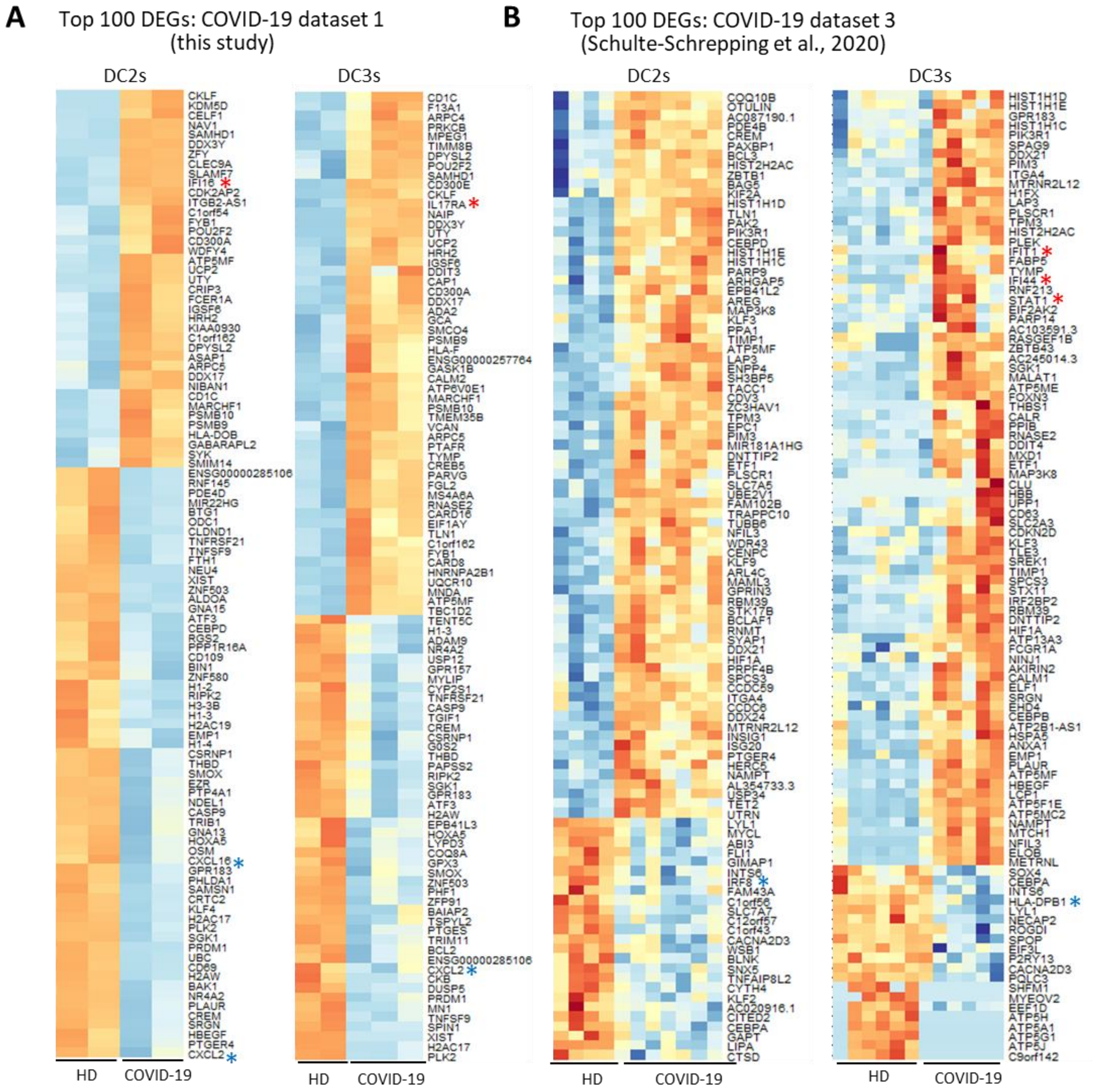

Supplementary Figure 5.

Heatmaps showing the top 100 DEGs for DC2s and DC3s comparing COVID-19 patients and HDs from (A) dataset 1 (DC2s: n=2 HDs, n=2 COVID-19; DC3s: n=2 HDs, n=3 COVID-19) and (B) dataset 3 (DC2s: n=4 HDs, n=7 COVID-19; DC3s: n=7 HDs, n=5 COVID-19). Selected up-regulated and down-regulated genes are indicated with an asterisk in red and blue, respectively. Ribosomal protein (RP) genes were removed from the top 100 DEGs (see Table S2 for full list of DEGs).

A GSEA with Blood Transcription Modules (BTM)

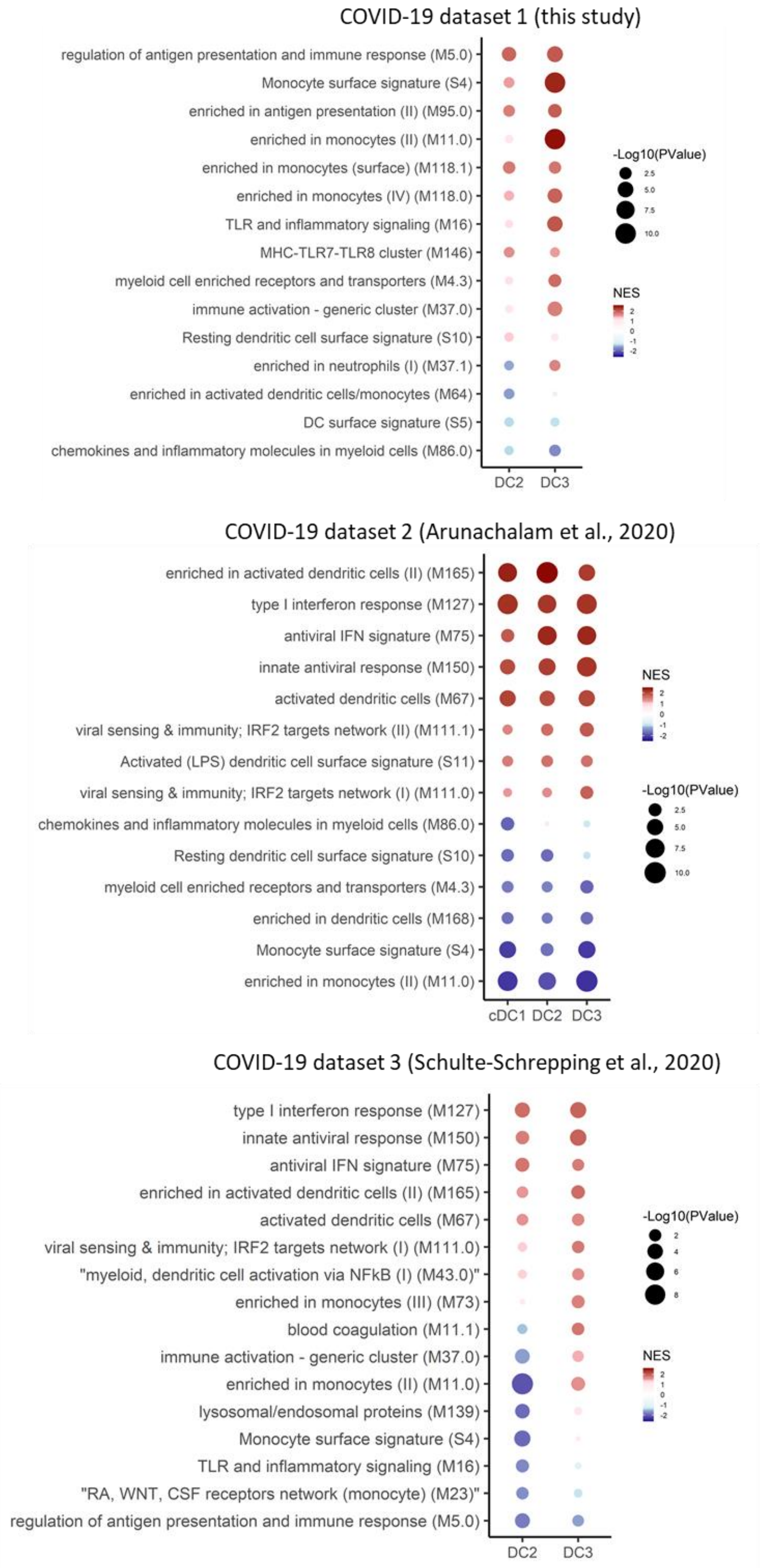

Supplementary Figure 6.

(A) GSEA of DEGs using the BTM collection: COVID-19 dataset 1 (upper panel), COVID-19 dataset 2 (middle panel) and COVID-19 dataset 3 (lower panel). The top 10 pathways in each cDC subset were selected and consolidated across all DC subsets in a single dot plot.

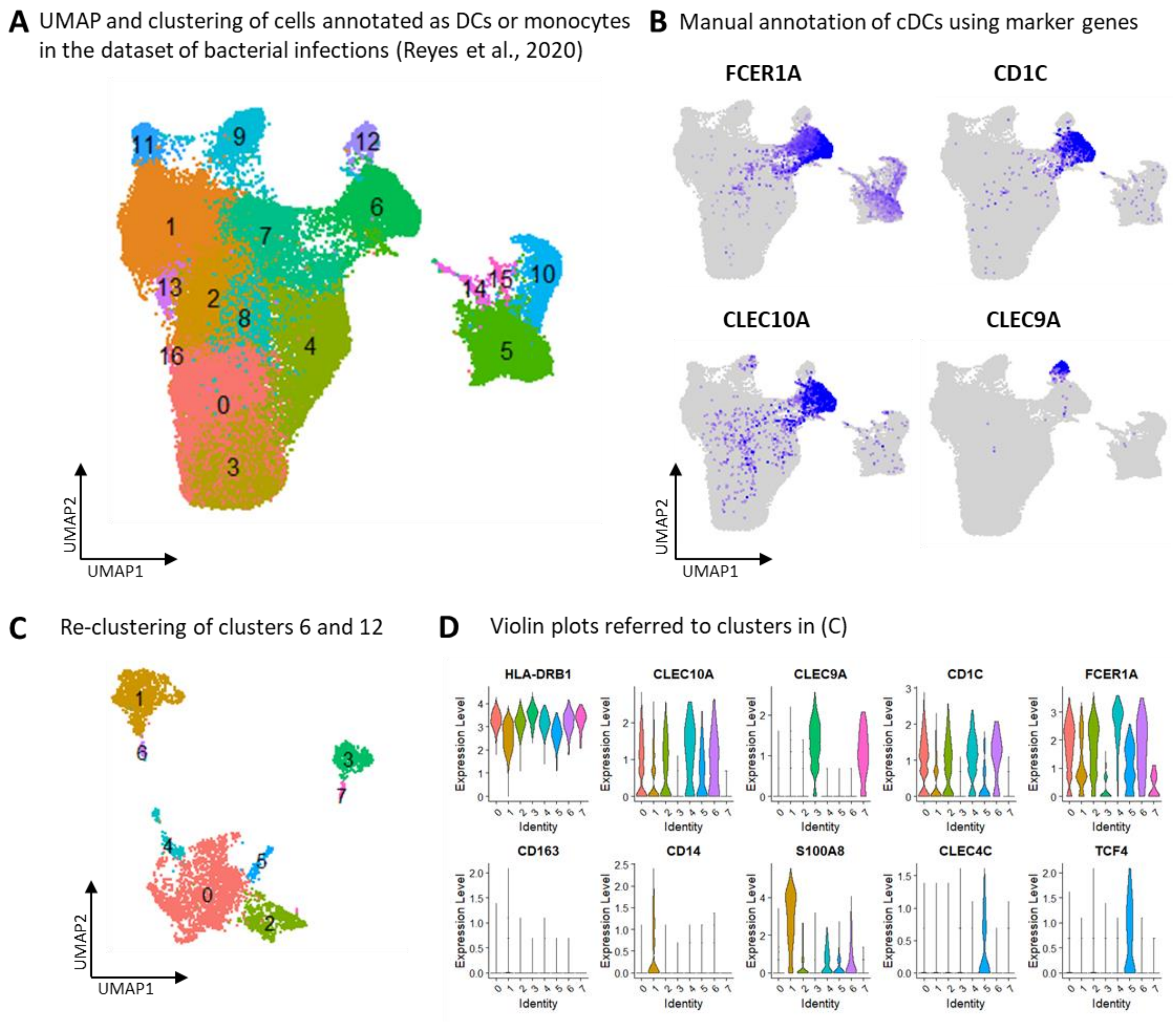

Supplementary Figure 7.

(A) UMAP and clustering of cells annotated as DCs or monocytes by the authors (dataset from Reyes et al., 2020). (B) Feature plots showing the expression levels of selected marker genes (FCER1A, CD1C, CLEC10A, CLEC9A) used to identify cDCs. (C) Re-clustering of clusters 6 and 12 corresponding to cDCs. (D) Violin plots referred to clusters in (C) showing expression levels of selected marker genes. Cluster 5, positive for CLEC4C and TCF4 was identified as contaminant and removed. All other clusters were re-clustered in a final iteration to clearly delineate cDC1, DC2 and DC3 subsets as shown in Figure 2A.

A Top 100 DEGs: dataset from Reyes et al., 2020 (bacterial infections)

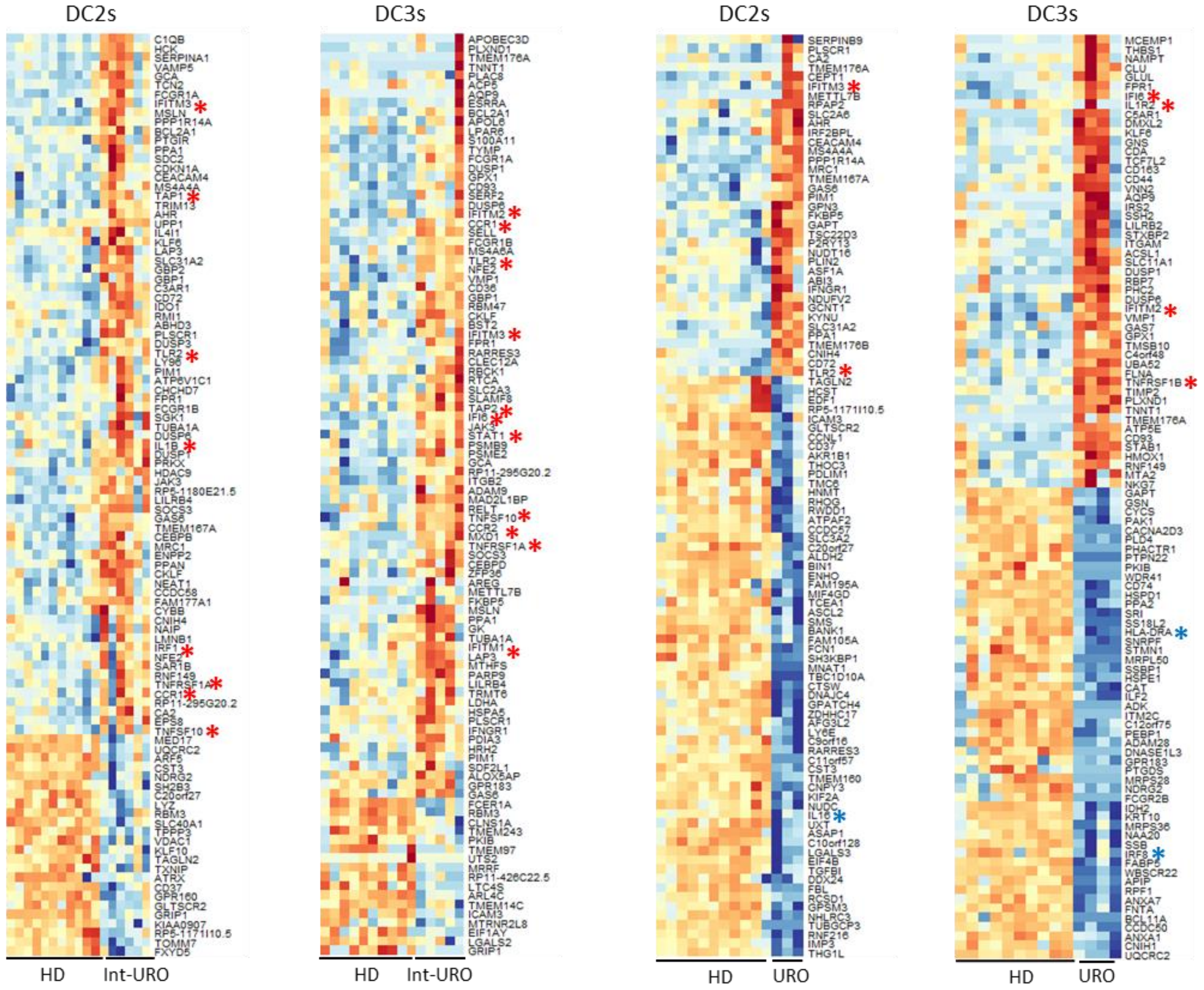

B Top 100 DEGs: dataset from Hao et al., 2020 (vaccine)

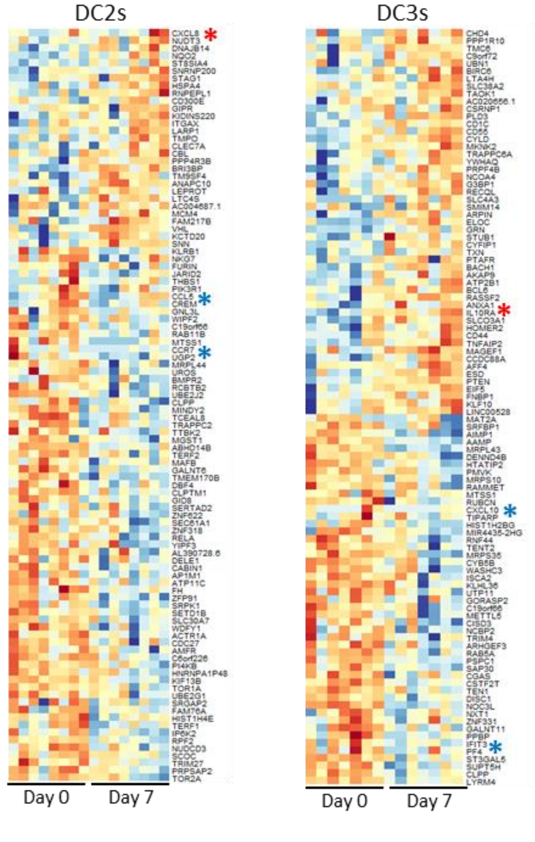

Supplementary Figure 8.

Heatmaps showing the top 100 DEGs for DC2s and DC3s from (A) Reyes et al. dataset (Int-URO vs HDs, DC2s: n=11 HDs, n=6 Int-URO; DC3s: n=10 HDs, n=5 Int-URO. URO vs HDs, DC2s: n=11 HDs, n=3 URO; DC3s: n=10 HDs, n=4 URO) and (B) Hao et al. dataset (DC2s: n=8 unvaccinated, n=8 vaccinated at day 7; DC3s: n=7 unvaccinated, n=7 vaccinated at day 7). Selected up-regulated and down-regulated genes are indicated with an asterisk in red and blue, respectively. Ribosomal protein (RP) genes were removed from the top 100 DEGs (see Table S4 for full list of DEGs). Int-URO, intermediate urosepsis. URO, urosepsis.

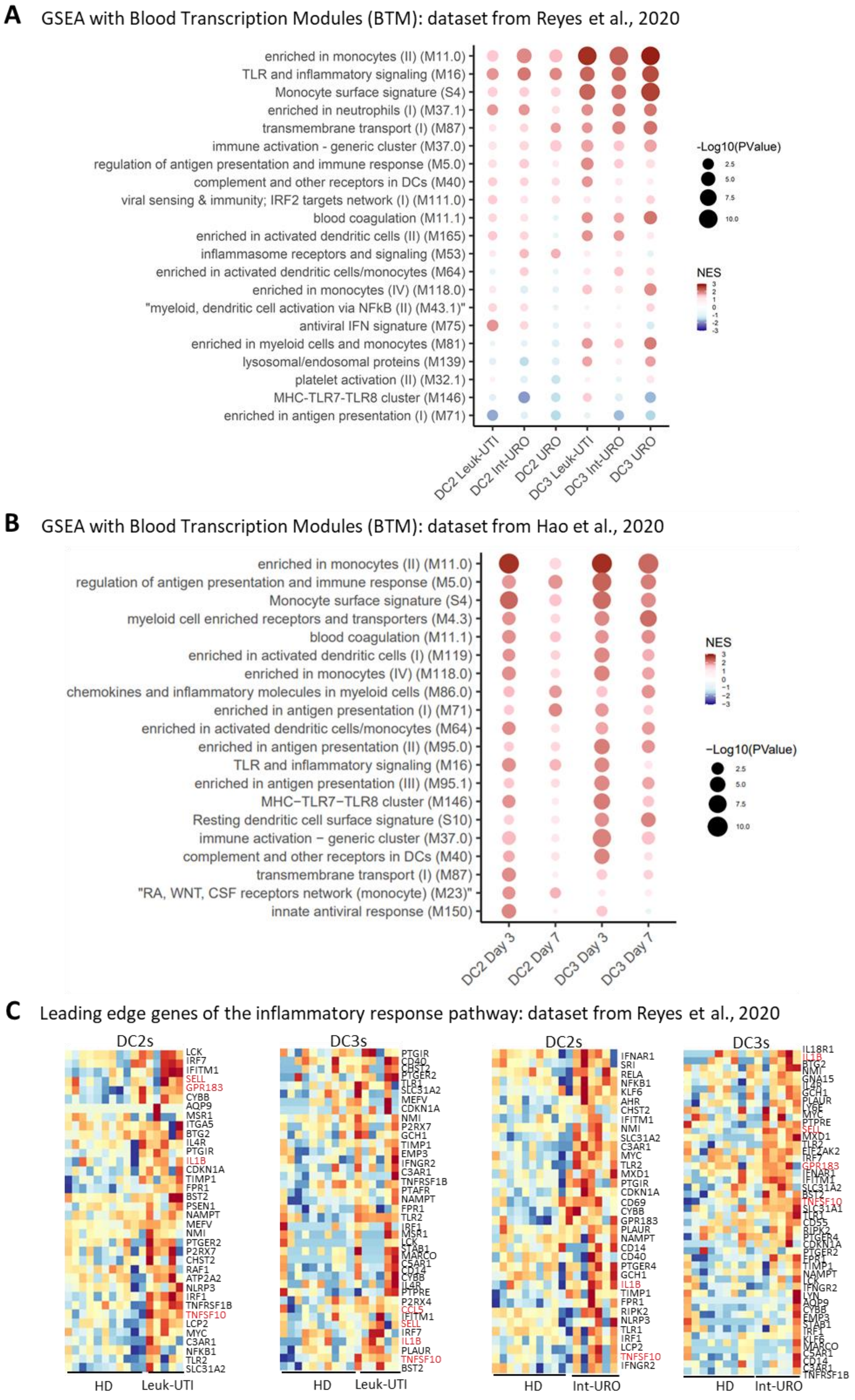
A GSEA with Blood Transcription Modules (BTM): dataset from Reyes et al., 2020
enriched in monocytes (II) (M11.0)
TLR and inflammatory signaling (M16)
Monocyte surface signature (S4)
enriched in neutrophils (I) (M37.1)
transmembrane transport (I) (M87)
immune activation - generic cluster (M37.0)
regulation of antigen presentation and immune response (M5.0)
complement and other receptors in DCs (M40)
viral sensing & immunity; IRF2 targets network (I) (M111.0)
blood coagulation (M11.1)
enriched in activated dendritic cells (II) (M165)
inflammasome receptors and signaling (M53)
enriched in activated dendritic cells/monocytes (M64)
enriched in monocytes (IV) (M118.0)
"myeloid, dendritic cell activation via NFkB (II) (M43.1)"
antiviral IFN signature (M75)
enriched in myeloid cells and monocytes (M81)
lysosomal/endosomal proteins (M139)
platelet activation (II) (M32.1)
MHC-TLR7-TLR8 cluster (M146)
enriched in antigen presentation (I) (M71)
DC2 Leuk-UTI
DC2 Int-URO
DC2 URO
DC3 Leuk-UTI
DC3 Int-URO
DC3 URO
-Log10(PValue)
2.5
5.0
7.5
10.0
NES
B GSEA with Blood Transcription Modules (BTM): dataset from Hao et al., 2020
enriched in monocytes (II) (M11.0)
regulation of antigen presentation and immune response (M5.0)
Monocyte surface signature (S4)
myeloid cell enriched receptors and transporters (M4.3)
blood coagulation (M11.1)
enriched in activated dendritic cells (I) (M119)
enriched in monocytes (IV) (M118.0)
chemokines and inflammatory molecules in myeloid cells (M86.0)
enriched in antigen presentation (I) (M71)
enriched in activated dendritic cells/monocytes (M64)
enriched in antigen presentation (II) (M95.0)
TLR and inflammatory signaling (M16)
enriched in antigen presentation (III) (M95.1)
MHC−TLR7−TLR8 cluster (M146)
Resting dendritic cell surface signature (S10)
immune activation − generic cluster (M37.0)
complement and other receptors in DCs (M40)
transmembrane transport (I) (M87)
"RA, WNT, CSF receptors network (monocyte) (M23)"
innate antiviral response (M150)
DC2 Day 3
DC2 Day 7
DC3 Day 3
DC3 Day 7
NES
−Log10(PValue)
2.5
5.0
7.5
10.0
C Leading edge genes of the inflammatory response pathway: dataset from Reyes et al., 2020
DC2s
DC3s
DC2s
DC3s
HD
Leuk-UTI
HD
Leuk-UTI
HD
Int-URO
HD
Int-URO

Supplementary Figure 9.

GSEA of DEGs using the BTM collection: (A) Reyes et al. dataset and (B) Hao et al. dataset. The top 10 pathways in each cDC subset were selected and consolidated across all DC subsets in a single dot plot. NES, normalized enrichment score. (C) Heatmaps showing GSEA leading edge genes of the inflammatory response pathway in the Reyes et al. dataset (Leuk-UTI vs HDs, DC2s: n=11 HDs, n=5 Leuk-UTI; DC3s: n=10 HDs, n=6 Leuk-UTI. Int-URO vs HDs, DC2s: n=11 HDs, n=6 Int-URO; DC3s: n=10 HDs, n=5 Int-URO). Selected genes are marked in red. Leuk-UTI, urinary tract infection with leukocytosis. Int-URO, intermediate urosepsis. URO, urosepsis.

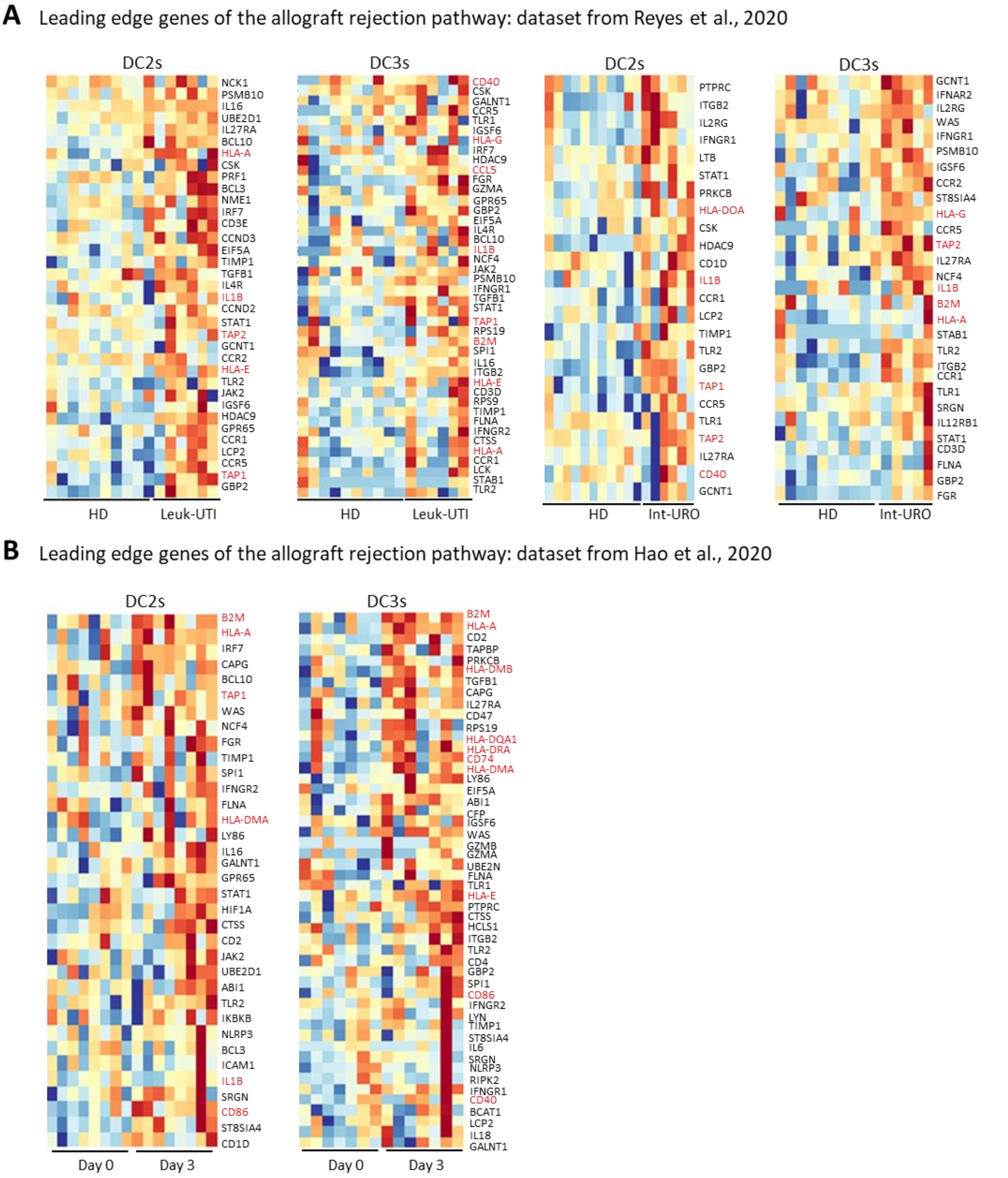

Supplementary Figure 10.

Heatmaps showing GSEA leading edge genes of the allograft rejection pathway in (A) Reyes et al. dataset (Leuk-UTI vs HDs, DC2s: n=11 HDs, n=5 Leuk-UTI; DC3s: n=10 HDs, n=6 Leuk-UTI. Int-URO vs HDs, DC2s: n=11 HDs, n=6 Int-URO; DC3s: n=10 HDs, n=5 Int-URO) and (B) Hao et al. dataset (Day 3 vs Day 0: DC2s: n=8 day 0, n=8 day 3; DC3s: n=7 day 0, n=7 day 3). Selected genes are marked in red. Leuk-UTI, urinary tract infection with leukocytosis. Int-URO, intermediate urosepsis. URO, urosepsis.

Table S1. Characteristics of COVID-19 patients and healthy donors (HD) enrolled in the study.

Table S2. DEGs between COVID-19 patients and healthy donors in each cDC subset from datasets 1, 2 and 3.

Table S3. BTM families used for GSEA.

Table S4. DEGs in each cDC subset from Reyes et al. and Hao et al. datasets.

Table S5. DEGs in DC3s vs DC2s COVID-19 patients vs HDs and DC3s vs DC2s Int-URO patients vs HDs.

Table S6. DEGs in DC2s and DC3s in COVID-19 patients with severe versus mild disease.
